# Glucose receptor deletion and engineering: impact on xylose sensing and utilization in *Saccharomyces cerevisiae*

**DOI:** 10.1093/femsyr/foaf040

**Published:** 2025-07-29

**Authors:** Bruna C Bolzico, Viktor C Persson, Raul N Comelli, Marie Gorwa-Grauslund

**Affiliations:** Grupo de Procesos Biológicos en Ingeniería Ambiental (GPBIA), Facultad de Ingeniería y Ciencias Hídricas (FICH), Universidad Nacional del Litoral (UNL), Ciudad Universitaria, CC 242 Paraje El Pozo, Santa Fe 3000, Argentina; Consejo Nacional de Investigaciones Científicas y Técnicas (CONICET), Ciudad Universitaria, CC 242 Paraje El Pozo, Santa Fe 3000, Argentina; Biotechnology and Applied Microbiology (BAM), Department of Process and Life Science Engineering (PLE), Lund University, Naturvetarvägen 22, SE-223 62, Lund, Sweden; Grupo de Procesos Biológicos en Ingeniería Ambiental (GPBIA), Facultad de Ingeniería y Ciencias Hídricas (FICH), Universidad Nacional del Litoral (UNL), Ciudad Universitaria, CC 242 Paraje El Pozo, Santa Fe 3000, Argentina; Consejo Nacional de Investigaciones Científicas y Técnicas (CONICET), Ciudad Universitaria, CC 242 Paraje El Pozo, Santa Fe 3000, Argentina; Biotechnology and Applied Microbiology (BAM), Department of Process and Life Science Engineering (PLE), Lund University, Naturvetarvägen 22, SE-223 62, Lund, Sweden

**Keywords:** xylose, saccharomyces cerevisiae, sugar signaling, Snf3p, Hxt2p, receptor engineering, GFP-based biosensor

## Abstract

Unlike glucose, the sub-optimal xylose utilization in recombinant *Saccharomyces cerevisiae* strains may stem from an unusual signaling response that is not adapted to detecting xylose as a fermentable substrate. We hypothesize that the membrane receptor Snf3p, known for sensing extracellular low glucose levels, may contribute to xylose recognition. To test this, we explored the effect of *SNF3* inactivation and overexpression by measuring the response of the *HXT2*p-GFP biosensor integrated into *S. cerevisiae* strains with heterogeneous xylose assimilation and metabolism capacities. We showed that the absence of *SNF3* effectively reduced *HXT2*p induction, while its overexpression improved signaling in the presence of xylose, suggesting the involvement of the receptor in the extracellular detection of this sugar. Although we attempted to engineer a xylose sensing system based on a chimeric receptor, its integration did not lead to considerable improvements in signal activation, indicating the need for further investigation. Finally, we showed that triggering the Snf3p pathway impacted xylose metabolism, with altered receptor levels prompting shifts in both biomass production and metabolite accumulation. Our findings suggest that understanding xylose sensing and its metabolic connection is essential for promoting more efficient xylose utilization in *S. cerevisiae*, a key step toward optimizing industrial bioprocesses.

## Teaser

The study reveals that the yeast *Saccharomyces cerevisiae*, known for fermenting glucose, also weakly senses xylose through a glucose receptor which may impact sugarÂ utilization.

## Introduction

*Saccharomyces cerevisiae* is the microorganism of choice for the implementation of bio-based ethanol production processes due to its efficient fermentation capabilities, its robustness and tolerance to industrial conditions, as well as its well-understood genetics (Hong and Nielsen 2012, Parapouli et al. 2020). Strains of this yeast have demonstrated success in fermenting glucose, sucrose, and maltose found in crops such as corn and sugarcane for the production first-generation (1G) bioethanol (Soccol et al. 2022). Replacing edible crops with alternative lignocellulosic feedstock, including agro-industrial wastes and dedicated energy crops, yields mixed glucose- and pentose-rich hydrolysates which can be used for second-generation (2G) ethanol production. However, the most prevalent pentose sugar in these hydrolysates is D-xylose, which is a non-natural substrate for wild-type *S. cerevisiae* strains and thus is not fermented (Hahn-Hägerdal et al. 2006). The economic feasibility for industrial exploitation of lignocellulosic materials for bioethanol production depends on the full conversion of C5 sugars from the hemicellulose fraction to maximize the final product titer. Xylose fermentation has been achieved in *S. cerevisiae* with good ethanol yields (Kuyper et al. 2005, Demeke et al. 2013, Cadete et al. 2016) by combining various metabolic and sugar transport engineering strategies and adaptive evolutionary approaches (reviewed by Jansen et al. 2017, Kwak and Jin 2017, Sharma and Arora 2020). However, the sugar consumption and ethanol production rates in recombinant xylose-fermenting *S. cerevisiae* strains are not reaching those obtained with glucose. Moreover, glucose preference of *S. cerevisiae* over other sugars available in mixed media causes a sequential use of the pentose sugars after glucose depletion. This pattern is a result of a complex regulatory mechanism known as glucose catabolite repression (Kayikci and Nielsen 2015) and of the kinetic properties of hexose transporters which favor glucose over xylose internalization (Lee et al. 2002, Apel et al. 2016).

The development of xylose-fermenting strains, based on the overexpression of heterologous xylose assimilation pathways such as xylose reductase/xylitol dehydrogenase (XR/XDH) from non-*Saccharomyces* yeasts and/or fungal or bacterial xylose isomerases (XI), seldom considers the regulatory effects that may affect the net performance of the strains on this foreign substrate. On glucose, *S. cerevisiae* has integrated signaling mechanisms that enable the yeast to rapidly respond to the presence of the sugar, thereby regulating and optimizing its catabolism (Rolland et al. 2002, Gancedo 2008). With unconventional substrates such as xylose, signaling interactions opposite to a fermentative behavior appear to be triggered (Matsushika et al. 2014, Brink et al. 2021). The xylose response in recombinant *S. cerevisiae* strains indicates starvation (Salusjärvi et al. 2006) as well as respiration rather than fermentation (Jin et al. 2004). Thus, once the heterologous xylose pathways have been introduced and *S. cerevisiae* strains has a functional capacity for xylose utilization, the regulatory mechanism for this sugar are not ideally tuned for fermentation—which may explain the suboptimal results observed on xylose.

**Figure 1.**
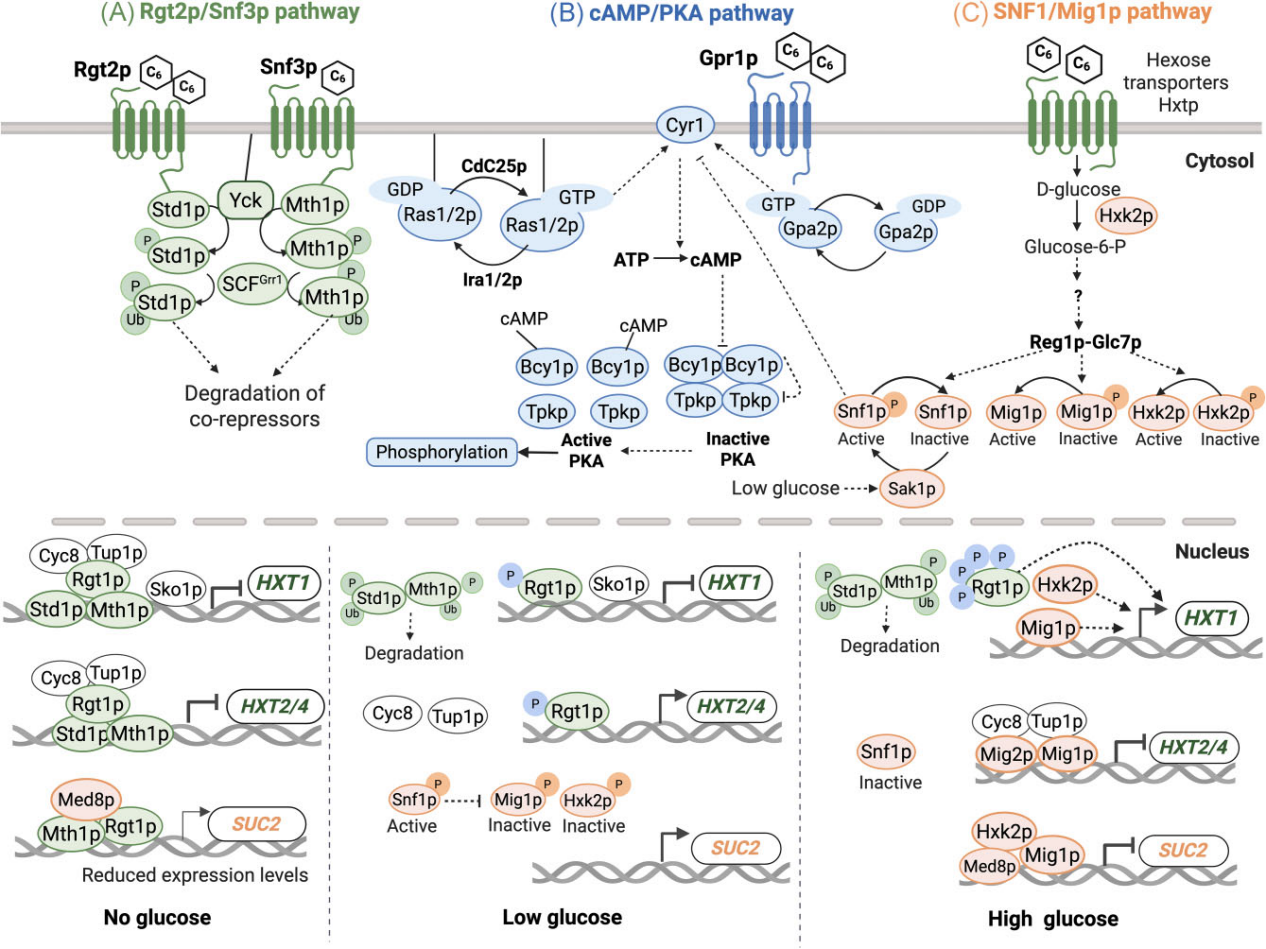
**Overview of glucose detection and key signaling mechanisms in the yeast *S. cerevisiae***. Three main signaling pathways are activated or repressed in the presence of glucose. (A) The *HXT*s transporter gene induction pathway is controlled by Snf3p and Rgt2p membrane receptors (green). Snf3p and Rgt2p sense low and high extracellular glucose levels, respectively, ultimately controlling glucose influx by regulating *HXT* transporter gene expression. (B) The glucose repression pathway mediated by SNF1/Mig1p (orange), controls the expression of genes involved in the metabolism of less preferred substrates. (C) The pathway leading to cAMP synthesis and activation of PKA (blue), which activates glycolytic enzymes, induces growth-related genes and represses stress genes. Transcription of representative genes (*HXT1, HXT2/4* and *SUC2*) is induced or inhibited under three different carbon source conditions: absence of glucose, low glucose (∼1 g L^−1^) and high glucose (>10 g L^−1^). The hammerhead represents repression and the arrowhead represents induction. Adapted from Osiro et al. (2018) and Brink et al. (2021).

The molecular mechanisms behind xylose sensing, or lack thereof, remain unclear. Previous evaluation of the effects of xylose on the three major glucose-signaling cascades (Fig. 1) has been performed by analyzing green fluorescent protein (GFP) expression from target promoters corresponding to the Snf3p/Rgt2p, SNF1/Mig1p, and cAMP/protein kinase A (PKA) sensing pathways (Brink et al. 2016, Osiro et al. 2018, Osiro et al. 2019). In XR/XDH strains, the induction response resembled the one triggered when *S. cerevisiae* is exposed to low glucose with, for instance, a de-repression of the extracellular invertase-encoding gene *SUC2* (Osiro et al. 2018, Fig. 1). In non-engineered *S. cerevisiae*, extracellular xylose triggered almost no response (Brink et al. 2016).

*S. cerevisiae* relies on two cell surface receptors, Snf3p and Rgt2p, to precisely control the gene expression of distinct hexose transporters of the *HXT* family (Fig. 1). These receptors sense the extracellular variations in glucose amounts and transduce the signal through a downstream cascade that degrades the co-repressors Mth1p and Std1p (Özcan et al. 1998, Kim et al. 2024). High glucose levels activate the Rgt2p receptor, which leads to the expression of the low-affinity hexose transporters, such as Hxt1p, while the expression of moderate-high affinity transporters, such as Hxt2p and Hxt4p, is induced via Snf3p which senses low levels of glucose (Özcan and Johnston 1995). Previous results have shown that the *HXT1*p biosensor was not induced by xylose, whereas the *HXT2p*/*4*p biosensors gave a heterogeneous signal in xylose cultures when glucose was absent (Brink et al. 2016). This indicated that some cells were responding to xylose while others were not (Brink et al. 2016). At the time, this variability in the signal was hypothesized to arise from stochastic uptake events, where a fraction of the population internalizes xylose through non-specific transporters, and endogenous xylose then triggers the induction response (Brink et al. 2016). However, an alternative hypothesis is that the Snf3p receptor can weakly detect and bind non-glucose sugars, such as xylose, leading to the transcription of *HXT2*/*4* genes. While some authors suggest that xylose is not recognized by Snf3p (Dietvorst et al. 2010), others claim that the receptor can trigger a minimal detection signal for this sugar (Wu et al. 2020). This discrepancy in findings regarding xylose recognition at the cell surface calls for additional testing.

From a strategic standpoint, the engineering of signaling pathways presents an attractive opportunity for improving microbial strains in a coordinated manner alongside metabolic pathway engineering. Therefore, we chose to further investigate the role of the Snf3p receptor pathway on xylose sensing by looking at the following questions: Does Snf3p give any signaling activity in response to extracellular xylose? Does activation of this signaling cascade correlate with restricted xylose utilization in an engineered pentose-fermenting strain? In parallel, we also assessed the potential of using the glucose membrane receptor pathway as a target for genetic engineering, with a goal of making xylose-specific chimeric receptors derived from Snf3p since activation of this pathway may provide improved pentose transport without necessitating constitutive expression of transporters.

## Materials and methods

### Strains, media, and general molecular biology techniques

The strains employed and generated in this work are detailed in Table 1. The *S. cerevisiae* lines integrating the *HXT2*p-y*EGFP* biosensor (TMB37 × 3 series) and the autofluorescence control strains (TMB37 × 1 series) were previously constructed by Brink et al. (2016) and Osiro et al. (2018), respectively. Both series include a background strain (TMB371X), a strain expressing a *GAL2*^N376F^ single mutated version of *GAL2* gene leading to improved xylose uptake ability (Farwick et al. 2014) (TMB372X), and one expressing the same *GAL2* mutated transporter gene together with the XR/XDH/XK xylose catabolic pathway genes and the *TAL1* and *TKL1* genes (TMB375X). Deletions and overexpression of the low-glucose receptor gene *SNF3* were executed in the negative control strains lacking the biosensor and in the reporter strains TMB37 × 3, which display a range of xylose uptake and metabolic capacities (Table 1). Yeasts were plated from −80°C culture stocks onto solid YPDYPD medium (10 g L^−1^ yeast extract, 20 g L^−1^ peptone, 20 g L^−1^ glucose, and 15 g L^−1^ agar) until colony growth. A single colony was pre-cultured in 5 mL YPD medium (10 g L^−1^ yeast extract, 20 g L^−1^ peptone, and 20 g L^−1^ glucose) for overnight growth at 30°C with orbital shaking at 180 rpm, followed by the lithium acetate transformation protocol (Gietz and Schiesl 2007). Transformants were selected on YPD plates supplemented with 200 μg mL^−1^ of G418 (Geneticin) and 100 μg mL^−1^ of ClonNAT (Nourseothricin) for the selection of Cas9 and gRNA plasmids expressing the *KanMX* and *NatMX6* markers, respectively. Both gene knockout and genomic integration of the genetic cassettes were guided by the CRISPR-Cas9 system developed by Stovicek et al. (2015). For the generation of the engineered strains, the yeasts were first transformed with the Cas9 plasmid (pCfB2312; Jessop-fabre et al. 2016).

**Table 1.** *S. cerevisiae* strains employed and constructed and their main genotypic characteristics.

Strain	Biosensor	Genotype	References
*Background strains*			
TMB3700		**W303-1A *TRP1 HIS3; ura3*::M3499 (*ADE2*)**	Brink et al. (2016)
TMB3711	**Control**	**TMB3700; can1::YIp211; SPB1/PBN1::YIp128**	Brink et al. (2016)
TMB3713	***HXT2*p**	**TMB3700; can1::YIp*GFP*-*HXT2*p; SPB1/PBN1::YIp128**	Brink et al. (2016)
*Xylose transporting strains*			
TMB3721	**Control**	**TMB3700; can1::YIp211; *SPB1*/*PBN1*::YIp128*GAL2*^N376F^**	Osiro et al. (2018)
TMB3723	***HXT2*p**	**TMB3700; can1::YIp*GFP-HXT2*p; *SPB1*/*PBN1*::YIp128*GAL2(N376F)***	Osiro et al. (2018)
*Xylose metabolizing strains*			
TMB3751	**Control**	**TMB3721; Vac17/MRC::*TKL*-*TAL; Chr*X-2/XI-5/XII-4::*XR*-*XDH*-*XK***	Osiro et al. (2018)
TMB3753	***HXT2*p**	**TMB3723; Vac17/MRC::*TKL*-*TAL; Chr*X-2/XI-5/XII-4::*XR*-*XDH*-*XK***	Osiro et al. (2018)
*SNF3 deletants*			
TMBB24	**Control**	**TMB3711; *snf3*Δ**	This study
TMBB11	**HXT2p**	**TMB3713; *snf3*Δ**	This study
TMBB25	**Control**	**TMB3721; *snf3*Δ**	This study
TMBB26	***HXT2*p**	**TMB3723; *snf3*Δ**	This study
TMBB27	**Control**	**TMB3751; *snf3*Δ**	This study
TMBB28	***HXT2*p**	**TMB3753; *snf3*Δ**	This study
*SNF3 overexpressing strains*			
TMBB35	**Control**	**TMB3711; *Chr*X4::*TEF1p-SNF3-GPM1*t**	This study
TMBB33	***HXT2*p**	**TMB3713; *Chr*X4::*TEF1p-SNF3-GPM1*t**	This study
TMBB34	***HXT2*p**	T**MB3753;*Chr*X4::*TEF1p-SNF3-GPM1*t**	This study
*Chimeric receptor strains*			
TMBB06	***HXT2*p**	**TMB3713; *Chr*XI-1::*SNF3*p-*GAL2mut*^N376Y/M435^trunc (Δ531)-*SNF3*tail-*SNF3*t**	This study
TMBB09	***HXT2*p**	**TMBB06; *snf3*Δ**	This study
TMBB12	***HXT2*p**	**TMB3713; *Chr*XI-1::*SNF3*p-*GAL2mut ^N^*^376Y/M435I^-*SNF3*t**	This study
TMBB13	***HXT2*p**	**TMB3713; *Chr*XI-1::*SNF3*p-*GAL2mut*^N376Y/M435I^ trunc (Δ531)-*SNF3*t**	This study
TMBB19	***HXT2*p**	**TMB3713; *Chr*XI-1::*SNF3*p-*GAL2mut*^N376Y/M435I^ trunc CT (Δ519)-*SNF3*t**	This study
*GFP-tagged strains*			
TMBB31		**TMB3711; pBB09**	This study
TMBB32		**TMB3711; pBB10**	This study

*Escherichia coli* NEB5α (New England Biolabs, MA, USA) competent cells were used for cloning, plasmid amplification, storage and preparation. Cells were grown at 37°C in LB medium (10 g L^−1^ tryptone, 5 g L^−1^ yeast extract and 5 g L^−1^ NaCl, pH 7.0) and the selection of positive transformants was achieved on plates (LB + 15 g L^−1^ agar) containing 50 μg mL^−1^ of ampicillin. The plasmids that were generated in the current study are listed in Supporting information Table S1.

All PCR products were purified using the GeneJET PCR Purification Kit (Thermo Fisher Scientific). All final constructs were confirmed by Sanger sequencing (Eurofins Genomics) and gene knockout was verified by colony PCR using *SNF3*-specific and flanking primers. Primers were purchased from Eurofins Genomics (Ebersberg, Germany); their sequences are provided in Supporting information Table S2.

Flow cytometry determinations and growth measurements were carried out in the defined YNB-KHPthalate (Yeast Nitrogen Base and Potassium Hydrogen Phthalate Buffer) medium supplemented with different concentrations of xylose and glucose, depending on the experiment.

### *SNF3* knockout

The *SNF3* open reading frame (ORF) was disrupted by CRISPR-Cas9-mediated homologous recombination. For this approach, a gRNA vector targeting the specific gene and a DNA disrupting fragment were constructed by PCR. The pCfB3042 plasmid (Jessop-Fabre et al. 2016) served as the backbone to amplify the new gRNA (pBBg5) using a unique reverse phosphorylated primer, BBg6_Rv, which binds to the SNR52 promoter, together with a forward phosphorylated primer, BBg5_Fw, which introduces specific 20 pb targeting sequence (Table S2). The 20 bp sgRNA was designed using CHOPCHOP (Montague et al. 2014). The purified PCR product was enzymatically digested with *DpnI*, and the linearized vector was ligated with T4 DNA ligase (Thermo Fisher Scientific), followed by *E. coli* transformation and plasmid recovery. The resulting gRNA vector, pBBg5, was Sanger sequenced and confirmed using primer LW_8 (Osiro et al. 2018). To repair the Cas9-generated double-strand break, a DNA donor fragment was PCR amplified using primer pairs BB01_Fw/BB02_Rv. The DNA template consisted of a 300pb fragment of the ampicillin resistance cassette (AmpR) flanked by 50pb sequences homologous to the regions up- and downstream of *SNF3* and the DNA break.

For the deletion of the sensor, strains carrying the Cas9 plasmid were transformed with 0.5 μg of the gRNA plasmid targeting *SNF3* along with 1 μg of the corresponding DNA repairing fragment. To ensure that the DNA donor was integrated into the gene locus and that the wildtype sensor was deleted, colony PCR was performed using the primers sets BB03_Fw/BB15_Rv and BB06_Fw/BB07_Rv (Table S2). The *snf3*Δ derivable strains were named TMBB09, TMBB11, TMBB24, TMBB25, TMBB26, TMBB27, and TMBB28 (refer to Table 1 for strain specifications).

### *SNF3* overexpression

The *SNF3* ORF was placed under a strong constitutive promoter and genomically integrated into biosensor strains at the X-4 locus (Jessop-Fabre et al. 2016). The *SNF3* overexpression cassette was constructed by amplifying *SNF3* gene from *S. cerevisiae W303* genomic DNA gene using primers BB56_Fw and BB57_Rv, introducing SfaI and MreI restriction sites, respectively. The insert was digested with MreI and SfaI restriction enzymes, subsequently purified, and ligated into the pUC57-ClosXI backbone between *TEF1*p and *GPM1*t, which had been digested with the same enzymes. The resulting plasmid, pBB11, was verified by colony PCR using primers BB03_Fw and BB04_Rv (see Table S2) and by Sanger sequencing. To enable integration of the overexpression cassette, two targeting fragments of approximately 500 bp corresponding to the upstream and downstream regions of the X-4 locus were PCR-amplified from W303 genomic DNA. Primers that added 55 bp tails homologous to the *TEF1* promoter and *GPM1* terminator of the *SNF3* cassette were used (see Table S2 for primer sequences). These homology tails matched the 5′ and 3′ ends of the linear cassette, facilitating double homologous recombination and genomic integration.

The *SNF3* overexpression cassette was integrated into *S. cerevisiae* wild-type and XR-XDH engineered strains. Strains harboring the Cas9 plasmid were co-transformed with 1 μg of the gRNA plasmid targeting the X-4 locus (pCfB3042); 0.5 μg of each targeting fragments homologous to the X-4 site and to the *SNF3* cassette described above; and 0.5 μg of the *TEF1*p-*SNF3*-*PGM1t* fragment excised from pBB11 using HindIII and KpnI enzymes. The correct integration was verified by colony PCR. The generated *SNF3*-overexpressing strains were named TMBB33, TMBB34 and TMBB35 (Table 1).

*Chimeric receptor design, construction, and integration*. The chimeric receptor Gal2pmut truncated-Snf3tail was built using a re-designed variant of the Gal2p transporter, this time incorporating two amino acids substitutions (N376Y and M435I) for increased xylose uptake (Rojas et al. 2021a). The transmembrane domains of the Gal2pmut transporter (amino acids 1 to 530) were then fused to the tail domain of the Snf3p receptor (amino acids 553 to 884). The chimeric gene expression cassette was ordered from Synbio Technologies (New Jersey, USA) and cloned into plasmid pBB01. *SNF3* promoter was used to control the expression of the chimera, and a region of 550 pb in length was selected upstream of the start codon of its ORF. For comparative purposes, three other constructs were tested in our analysis. These included the Gal2pmut^N376Y/M435I^ transporter (amino acids 1 to 574); the truncated version of Gal2pmut^N376Y/M435I^ present in the chimera but lacking the sensing domain (Gal2mut trunc; amino acids 1 to 530); and the C-terminal fully deleted Gal2pmut^N376Y/M435I^ (Gal2mut trunc CT; amino acids 1 to 519).

The strain carrying the chimera, TMBB06, was constructed using the *HXT2*p-y*EGFP* biosensor strain TMB3713 (Brink et al. 2016) as backbone. The *SNF3*p-*GAL2mut*^N376Y/M435I^ trunc-*SNF3*tail-*SNF3*t genetic construct was inserted into the intergenic locus XI-1 (ChrXI; MNN4/PTK1) (Jessop-Fabre et al. 2016) of the mentioned background strain. To carry out the gene insertion, the cassette was excised from pBB01 using *Asc*I/*Sbf*I/*Pdm*I and the insert flanked by *Asc*I/*Sbf*I ends was gel-purified and subsequently ligated with pCfB3036 backbone (Jessop-Fabre et al. 2016). To allow the ligation of the chimeric gene within the integration sites specified in the backbone, the integrative plasmid pCfB3036 was PCR amplified using primers 35–36_Fw/36_Rv introducing the *Asc*I site, followed by *Asc*I/*Sbf*I digestion and purification. The expression cassette and the linear vector were successfully ligated and the resulting plasmid pBB01INT was isolated from *E. coli* and verified by Sanger sequencing and colony PCR (Table S2).

The plasmid pBB01INT was used as template to construct *GAL2mut* genetic variants by restriction enzyme cloning. A fragment of 371 pb containing the missing part of the Gal2pmut C-terminal domain was amplified using the primers GAL2_MfeI_Fw/GAL2_NcoI_Rv from YIplac128-GAL2_N376F (Osiro et al. 2018), followed by digestion with NcoI and MfeI and the fragment was then ligated to pBB01INT backbone cleaved with the same restriction enzymes. This yielded plasmid pBB03INT (*GAL2mut* transporter). Similarly, to construct pBB04INT (*GAL2mut* trunc) and pBB05INT (*GAL2mut* trunc CT), two fragments corresponding to each part of the truncation were PCR amplified with the same forward primer, GAL2_BglII_Fw, and different reverse primers, GAL2trunc_NcoI_Rv and GAL2trunc_full_NcoI_Rv, respectively, cleaved with BglII/NcoI and subsequently ligated with the digested pBB01INT using the same restriction sites.

The chimeric receptors expressing-strains, TMBB06 and TMBB07, were generated by co-transformation of TMB3713 and TMB3711, respectively, with 1 *μ*g of the expression cassette flanked by targeting fragments obtained by linearization of pBB01INT with *Not*I. In addition, 1* μ*g of the respective gRNA helper vector pBBg12 was used. The plasmid was constructed in this work using the S. *cerevisiae* W303 strain target sequence (5´-GCGGTGCACGGATTTCAGCA-3´) due to its non-identical XI-1 intergenic region compared to that present in the CEN.PK background, for which the CRISPR-Cas9 system was designed (Jessop-Fabre et al. 2016). PCR amplification with phosphorylated primers BBg12_Fw and BBg6_Rv, recircularization of the amplicon with T4 DNA ligase (Thermo Fisher Scientific) and transformation of *E. coli* competent cells were used to generate the new gRNA using the pCfB3042 backbone plasmid. After yeast transformation and selection, the correct integration of the chimeric expression cassette into the defined site in the genome was confirmed by yeast colony PCR using primers that anneal outside the recombination site (XI-1_Ver_Int_Rv), as well as an internal forward primer that binds to the *SNF3* tail region (Int_SNF3_tail_Ver_F). The strains harboring the mutated Gal2pmut^N376Y/M435I^ transporter (TMBB12), the truncated version of this transporter used in the chimera construct (TMBB13), and the fully truncated version with the entire C-terminus deleted (TMBB19) were generated in the same manner (Table 1).

### Flow cytometry

A similar experimental design as described in Brink et al. (2016) was used for flow cytometry measurements with the following modifications: the MACSquant VYB flow cytometer (Miltenyi Biotec, Germany) equipped with the B1 (525/50 nm) and B2 (585/40 nm) filters was used to quantify emitted fluorescence and 20 000 events per sample were collected. Single cell fluorescence intensity (FI) analysis was performed in each culture condition consisting of 250 mL baffled flasks containing YNB-KHPhtalate with either no sugar, glucose 1 g L^−1^, xylose 50 g L^−1^, or glucose 40 g L^−1^, at a starting OD_620nm_ of 0.4. Samples were taken at 0 h (immediately after strain inoculation) and at 6 h, diluted if necessary with phosphate-buffered saline (PBS) at pH 7.4 to an OD_620nm_ between 0.20 and 0.40, treated with 10 μg mL^−1^ propidium iodine (PI), and analyzed in the flow cytometer at a rate of 25 µL min^−1^. Prior to conducting the analysis, an initial pre-culture of single colonies of GFP reporter and autofluorescence strains was performed in 5 mL Falcon tubes with YNB-KHPhtalate glucose 20 g L^−1^ to reach sufficient biomass (10 h) at 30°C and 180 rpm. The cells were then transferred to 250 mL flasks containing 25 mL of YNB-KHPhtalate with 40 g L^−1^ glucose, i.e. the established *HXT2*p biosensor repression condition according to Osiro et. al. (2018). The flasks were inoculated with an initial OD_620nm_ of 0.05 and grown overnight for 12 h. The repression cultivation was done to test the biosensor response in the different media conditions at a starting point with minimal fluorescence signal.

For non-biosensor strains (see control strains in Table 1), autofluorescence signals were determined under repression and induction conditions for the *HXT2* gene, 40 g L^−1^ and 1 g L^−1^ glucose, respectively, as well as in 50 g L^−1^ xylose and no carbon source cultures.

Flow cytometric data from two independent biological replicates were collected and analyzed using the FlowJo v10 software (BD Life sciences). The fold change between the FI of the final sample (6 h) and the initial sample (0 h) was calculated to follow the induction/repression response of the biosensor to the different conditions studied. When dual GFP-populations appeared during xylose cultivation, the dot plots corresponding to FSC-A vs GFP-A were manually gated to separate the subpopulation with lower (on the left) and higher (on the right) FI. See Fig. S1 in Supporting information for an example on how low-FI and high-FI populations were separated from a heterogeneous set of cells on xylose cultures. The distribution of each population was calculated as percentage along with the FI geometric means. Additionally, for the induced subpopulation the FI values at 6 h were correlated with those at 0 h to calculate the fold change of the induced peak.

### RNA isolation and RT-qPCR analysis

Cells were cultivated under the same conditions as described above for flow cytometry analysis, with samples collected at 15 and 45 min after inoculation in medium containing either 1 g L^−1^ glucose or 50 g L^−1^ xylose. Culture samples were subjected to cold methanol quenching (−80°C) and centrifuged at 0°C at 1800 rpm. The cell pellets were then washed with cold RNase-free water and stored at − 80°C until RNA extraction. Total RNA was purified using the MasterPure™ Yeast RNA Purification Kit (Biosearch Technologies), which includes treatment with DNase I to remove genomic DNA. For cDNA synthesis, 0.4 µg of total RNA from each sample was reverse transcribed using the PrimeScript™ RT Master Mix kit (Takara Bio). RT-qPCR reactions were carried out using the TB Green® Premix Ex Taq™ II (Tli RNaseH Plus), ROX Plus kit (Takara Bio), on a QuantStudio™ 5 Real-Time PCR System (Thermo Fisher Scientific). *ACT1* was used as the reference gene, as it has previously demonstrated high expression stability under similar conditions (Brink et al. 2016), while *HXT2* was used as the target gene. Primer sequences are listed in Table S2. For each sample, RNA extract without reverse transcriptase was included in the reactions to verify that amplification was specific to cDNA and not due to residual genomic DNA, along with a no-template control. The following thermal cycling conditions were used for both genes: initial denaturation at 96°C, 30 s; 40 cycles of 96°C, 5 s and 60°C, 30 s; followed by melting curve analysis starting at 60°C, 20 s, ramping to 95°C at 0.15°C/s, and holding at 95°C for 1 s. Data processing, including Cq value determination and melting curve analysis, was performed using Design & Analysis 2 (DA2) software version 2.8.0 (Thermo Fisher Scientific). Amplification efficiencies of the primers were determined using standard curves. Relative gene expression changes and statistical analyses were performed using the R package “rtpcr”, specifically the qpcrANOVARE function, which incorporates efficiency correction and allows multifactorial ANOVA analysis for qPCR data (Ganger et al. 2017). Each condition was analyzed in biological triplicates with technical replicates to account for technical variability.

### Growth experiments and analytical determinations

Cells were cultivated in 25 mL YNB-X50 in 250 mL baffled shake flasks with an orbital shaker set to 280 RPM and 30°C. Samples for optical density and metabolite analysis were taken at 0 h, 8 h, 24 h, 32 h, 48 h, 55 h, 61 h, 73 h, 79 h, 144 h, and 170 h in biological duplicates. Optical density was determined at 620 nm using an UltroSpec 2100 Pro spectrophotometer (Amersham Biosciences, Buckinghamshire, United Kingdom), and cell dry weight was measured at the end of the cultivation using 0.4 µm filtration in three technical replicates. Extracellular metabolite concentrations were determined using a Waters 2414 refractive index (RI) detector and a Phenomenex Rezex ROA-Organic Acid H + column (8%) in a Waters HPLC system (Milford, USA) running isocratically at 0.6 mL min^−1^ with a mobile phase of 5 mM H_2_SO_4_ at 60°C.

### GFP-tagged constructs and fluorescence microscopy

To analyze subcellular localization, GFP was fused to the C-terminus of the *GAL2*mut transporter and the chimeric construct through homologous recombination in *S. cerevisiae*, using PCR amplified fragments with homologous overhangs. yEGFP ORF was amplified from the YIpGFP plasmid (Brink et al. 2016) using forward primers containing 50 bp of homology either to the 3’ end of the *SNF3* tail (for the chimeric construct) or to the 3’ end of the *GAL2*mut transporter. All primers used for the fusion are listed in Table S2. To allow in vivo plasmid assembly via homologous recombination, the resulting overlapping fragments were co-transformed with the HindIII/EcoRI-linearized pRS42N multicopy plasmid into *S. cerevisiae* TMB3711 strain. After selection, plasmids were extracted from yeast using the Zymoprep Yeast Plasmid Miniprep Kit (Zymo Research) and subsequently propagated in *E. coli* DH5α for amplification and storage. Correct assembly of the vectors was confirmed through whole-plasmid sequencing (Eurofins Genomics). The resulting plasmids, named pBB07 and pBB08, express the chimeric construct-GFP and the *GAL2*mut-GFP transporter, respectively, under the control of the *SNF3* promoter. To overcome the very low fluorescence signal, the *SNF3* promoter was replaced with the strong constitutive *TDH3* promoter. For promoter replacement, an approximately 1600 bp *TDH3p-GAL2* fragment was amplified from YIplac128 using primers GAL2_BglII_Rv and TDH3p_SalI_Fw and subsequently digested with BglII and SalI enzymes. The insert was then purified and cloned into the pBB07 and pBB08 backbones, previously digested with the same enzymes. Following ligation and transformation into *E. coli* competent cells, plasmids were recovered and verified by colony PCR and whole-plasmid sequencing. The final constructs, named pBB09 and pBB10, were then transformed into *S. cerevisiae* TMB3711 for fluorescence microscopy analysis, yielding TMBB31 and TMBB32 strains, respectively.

For fluorescence microscopy, *S. cerevisiae* TMBB31 and TMBB32 strains expressing the GFP constructs were cultivated overnight in 5 mL YNB medium supplemented with 20 g L^−1^ glucose and 100 μg mL^−1^ ClonNAT. Cells were then washed, and an inoculum was prepared. Fresh YNB medium containing 50 g L^−1^ xylose or 20 g L^−1^ glucose was inoculated at an initial OD_620nm_ between 1.5 and 2. Cell cultures were incubated at 30°C and 180 rpm for 5 h. GFP fluorescence was then detected using a Leica SP8 DLS confocal microscope. Images were collected in separate channels for phase contrast and fluorescence and analyzed with Fiji software.

## Results

### Is Snf3p involved in extracellular xylose sensing?

To clarify the role of the Snf3p receptor in xylose sensing, we performed *SNF3* deletion and *SNF3* overexpression in *S. cerevisiae* biosensor strains, which allowed measurement of *HXT2* induction/repression responses via flow cytometry.

The Snf3p receptor was deleted in three different types of biosensor strains in order to gain a holistic understanding of the phenomenon: (1) in strain TMB3713 carrying only the GFP-based *HXT2*p biosensor, (2) in strain TMB3723 carrying the biosensor as well as a Gal2mut transporter with xylose specificity, and 3) in strain TMB3753 carrying the biosensor, the Gal2pmut transporter and a recombinant XR/XDH pathway for xylose catabolism (Brink et al. 2016, Osiro et al. 2018). This yielded *SNF3* deletant strains TMBB11, TMBB26, and TMBB28, respectively (Table 1). The deletion was also performed in the respective control strains (i.e. isogenic strains with no biosensor cassette) to assess the level of autofluorescence and uncover any potential differences in cell size or morphology that could influence the fluorescence signals (refer to Supporting information, Fig. S2). This resulted in strains TMBB24, TMBB25, TMBB27, respectively (Table 1).

**Figure 2.**
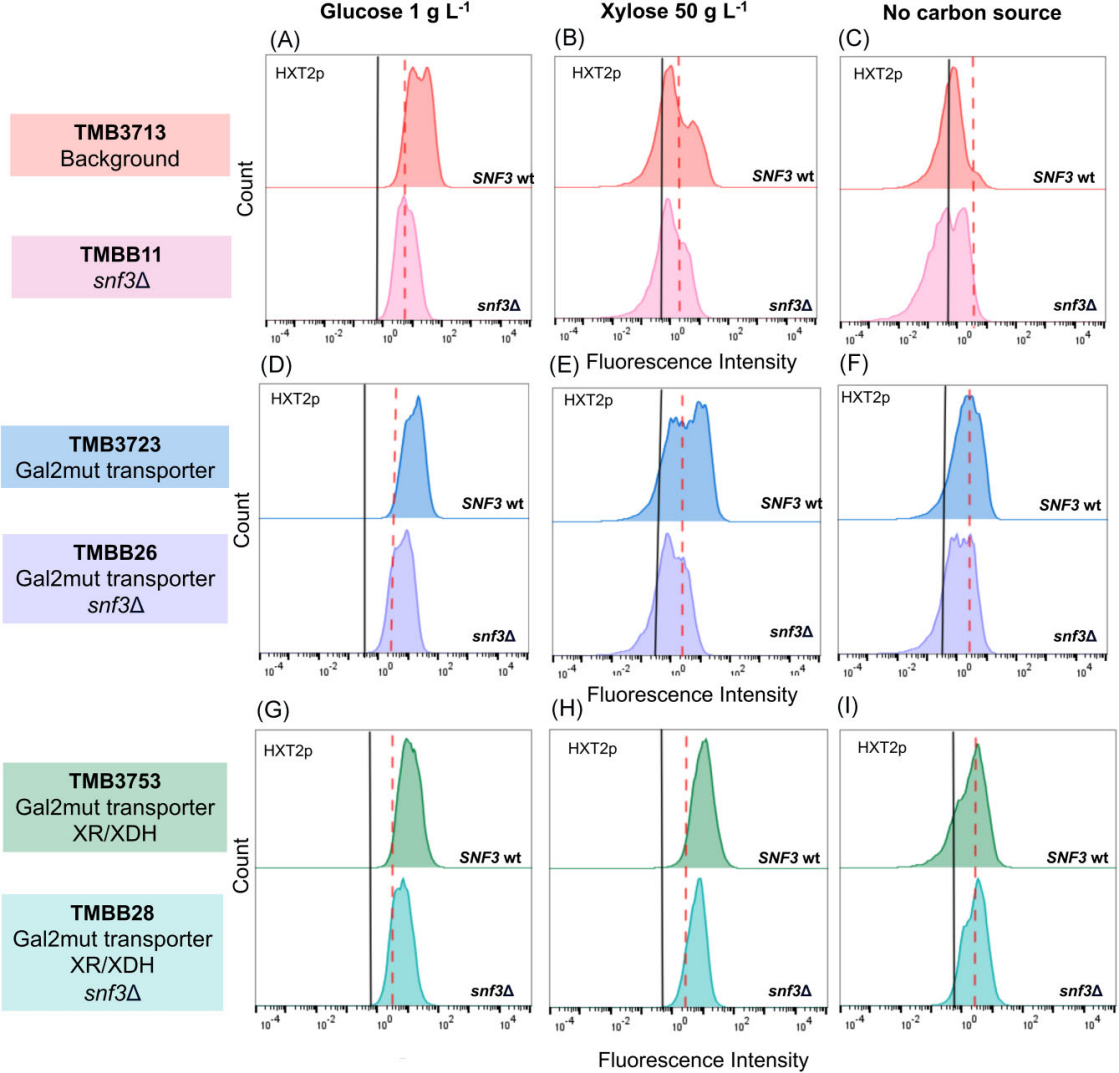
***HXT2p-*GFP expression is decreased in high xylose and low glucose cultures when *SNF3* is inactivated**. The fluorescence profile histograms of *HXT2*p biosensor strains are shown for TMB3713 (A–C), TMB3723 (D–F) and TMB3753 (G–I) together with their *snf3*Δ derivatives: TMBB11, TMBB26, and TMBB28, respectively. The GFP response was tested on cells grown on glucose 1 g L^−1^ (the biosensor induction condition), xylose 50 g L^−1^, and YNB alone (no carbon source) after 6 h. The red dotted line indicates the repression state of the corresponding TMB37 × 3 strain at 0 h for each of the conditions. The autofluorescence of the negative corresponding control strain (TMB37 × 1) is indicated by the black line. Experiments were performed in biological duplicates in shake flasks, representative histograms are shown.

The response of the *HXT2*p biosensor was assayed at 1 g L^−1^ glucose (established induction condition), 50 g L^−1^ xylose, and without carbon source in *SNF3*-wildtype strains and their respective *SNF3* deletion counterparts (Fig. 2). The data, including geometric means of fluorescent intensities and population distributions, was numerically summarized (Table S3-S4). Prior to the experiment, the biosensor was repressed using 40 g L^−1^ glucose, since this had previously been shown to increase the dynamic range of certain biosensors (Brink et al. 2016, Osiro et al. 2018). Despite this, the *HXT2* promoter was not fully repressed at the start of our experiments, as reflected by the difference in fluorescence between the repression condition (red dashed line) and the autofluorescent control strain (black line) (Fig. 2). The reduced dynamic range was nevertheless deemed acceptable, especially considering previous reports of similar difficulties in fully repressing the *HXT2*p biosensor (Brink et al. 2016, Osiro et al. 2018).

Upon addition of xylose to the repressed cells of the *SNF3*-intact strain (TMB3713), two sub-populations were obtained, in agreement with previous results reported by Brink et al. (2016). In the induced sub-population, representing ca. 30% of the total population, a 3.4 ± 0.9-fold increase in biosensor fluorescent intensity was observed (Fig. 2B). This level of induction was comparable to the one detected under low glucose conditions (4.1 ± 0.4-fold increase; Fig. 2A), indicating that the Snf3p receptor was active on xylose. The induction was not observed in the control condition lacking carbon sources, which also demonstrated that the subpopulation induction was xylose-specific (Fig. 2C).

After the deletion of *SNF3* (TMBB11), the induction of *HXT2*p was no longer observed on low glucose concentrations (Fig. 2A), as expected. On xylose, the same phenotype was also observed (Fig. 2B), strengthening the hypothesis that the Snf3p receptor plays a direct role in the sensing of extracellular xylose.

It has previously been suggested that the observed induction of *HXT2* gene could arise from alternative endogenous signaling routes via xylose internalization into the cells rather than from direct sensing via Snf3p (Brink et al. 2016). Therefore, control strains carrying a dedicated xylose transporter with and without *SNF3* gene deletion were assayed as well. In these strains with enhanced xylose uptake capacity, the induction only appeared on xylose when *SNF3* was intact (Fig. 2E), confirming the hypothesized sensing role of Snf3p on xylose. The addition of the transporter protein (Gal2p with N376F substitution; TMB3723) did increase the fraction of induced cells in the population, with an approximately 1.5-fold increase in the fluorescence signal of the biosensor (Fig. 2D-F and Table S3); however, analysis of flow cytometry data showed that this apparent higher signal was probably caused by a rise in cell size as the same increment factor was obtained in the FSC-A parameter when comparing TMB3713 and TMB3723 strains (see Table S5 for normalized data).

Osiro and colleagues previously demonstrated that a strain carrying both the Gal2pmut transporter and a heterologous xylose utilization pathway (TMB3753) showed a single population of induced *HXT2*p-GFP, as compared to the two sub-populations observed in the strain carrying only the Gal2mut transporter (Osiro et al. 2018). We confirmed this result and the fact that the population exhibited a fluorescence similar to the induced population of the non-engineered strain (Fig. 2H) and a general sugar signaling response pattern as the one activated by low glucose (Osiro et al. 2018). Upon *SNF3* gene deletion, the induction of *HXT2*p was reduced 1.5 fold (TMBB28; Fig. 2G–I & Table S3). This indicates that although full induction of *HXT2* relies on the Snf3p receptor, cells able to metabolize xylose can achieve induction or derepression via other intracellular signals downstream of the receptor, such as the phosphorylation of Mig1p by the SNF1 complex (Fig. 1).

**Figure 3.**
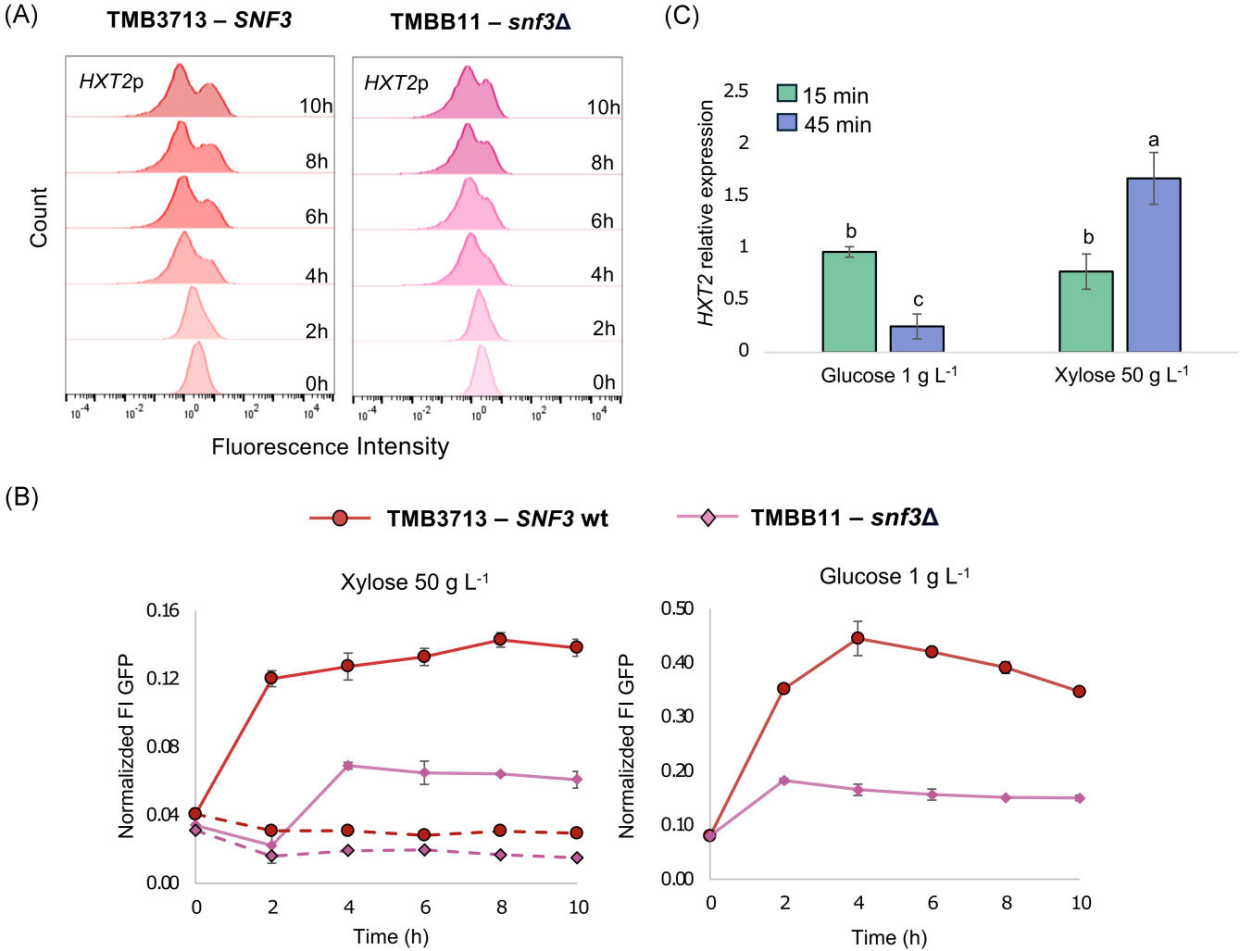
**Changes in *HXT2*p-GFP fluorescence and *HXT2* expression over time in response to high xylose and low glucose**. (A) Fluorescence histogram plots for TMB3713 (*SNF3* wild-type) and TMBB11 (*SNF3* deletant) strains initially display uniform populations on xylose, which then shift towards population heterogeneity. Histograms show representative results from biological replicates. (B) *SNF3* inactivation causes *HXT2*p-GFP signals to decrease. Normalized Fluorescence Intensity values to cell size of the induced population (solid lines) and the total population (dotted lines) plotted against time. Strains were cultivated on YNB flasks with xylose 50 g L^−1^ or glucose 1 g L^−1^ and samples for flow cytometry analysis were taken at several time points. The error bars indicate the standard deviation from two biological replicates. C) *HXT2* is expressed in low glucose and high xylose in TMB3713 strain, although the temporal expression profiles differ between these conditions. Results are based on RT-qPCR analysis of relative expression levels. The error bars represent the standard error from three independent biological triplicates. Different letters indicate statistically significant differences between conditions, as determined by a one-way analysis of variance (ANOVA) followed by a post hoc test (Tukey's HSD).

To rule out any delayed effects on the biosensor induction after *SNF3* knockout, the signaling response to xylose was monitored over time in strains TMB3713 (*SNF3* wild-type) and TMBB11 (*snf3* deletant) carrying only the GFP-based *HXT2*p biosensor (Fig. 3A and 3B). The lower FI displayed after *SNF3* deletion (TMBB11) was maintained over the course of 10 h, and no increase in the GFP intensity of the *HXT2*p biosensor was observed during this period of cultivation on xylose (Fig. 3B, left panel). Comparable results were obtained in the low glucose control condition after *SNF3* deletion (Fig. 3B, right panel). In addition, the mentioned changes remained consistent following the normalization of the FI signals in relation to cell size (see Table S5 for the normalized data). Notably, although two subpopulations did appear eventually regardless of Snf3p activation, in the *snf3*Δ strain the manifestation of the high-FI population was significantly delayed compared to the wild-type strain, and below the initial repressed level (Fig. 3B). Taken together, we can effectively argue that the decline in *HXT2*p-GFP fluorescence seen on xylose was caused by an incomplete Snf3p signaling transduction pathway rather than decreased cell size or delayed signal induction.

To confirm that the *HXT2*p-GFP biosensor signal correlates with native transcriptional regulation of *HXT2* gene under the different sugar conditions, RT-qPCR was performed to measure endogenous *HXT2* mRNA levels in the TMB3713 strain. *HXT2* expression was elevated in cultures exposed to high xylose and low glucose (Fig. 3C), in agreement with the flow cytometry data. *HXT2* transcript levels also decreased over time in the presence of low glucose (Fig. 3C), which is consistent with the transient GFP signal shown in Fig. 3B (right panel). This indicates active glucose uptake and metabolism in TMB3713 strain, which likely triggers rapid adaptation and subsequent downregulation of *HXT2* gene upon glucose depletion. In contrast, under xylose conditions—where this strain cannot actively metabolize the sugar—*HXT2* expression increased progressively over time (Fig. 3C). This suggests that the continuous presence of extracellular xylose may engage the Snf3p sensing pathway, thereby maintaining *HXT2* transcription despite the absence of significant metabolic activity.

**Figure 4.**
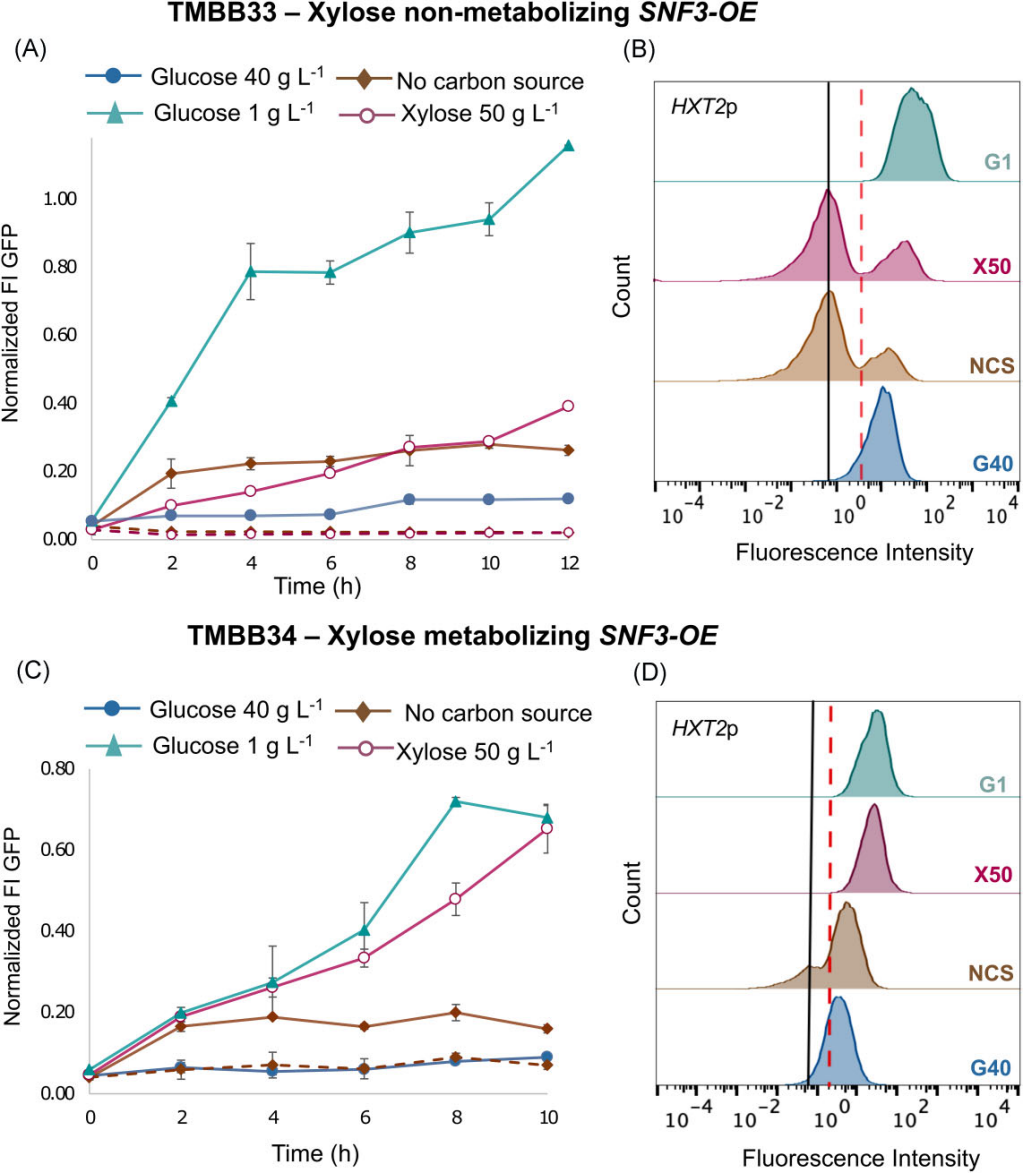
***SNF3* overexpression generates a constitutive *HXT2*p signal, which is further amplified under low glucose and high xylose conditions**. Flow cytometry analysis is shown for TMBB33 (TMB3713; *SNF3*-OE) in panels A and B and for TMBB34 (TBM3753; *SNF3*-OE) in panels C and D. Time-course plots show the normalized fluorescence intensity (relative to cell size) of the *HXT2*p biosensor for strains cultivated in YNB with different carbon sources and without sugar addition. Dotted lines indicate the average fluorescence of the total cell population when two distinct subpopulations appear. Histograms show the distribution of fluorescence intensity per event in the cell samples at 12 h of cultivation for panel B and 10 h for panel C. The black lines represent the autofluorescence levels of the control strain (TMBB35; *SNF3*-OE), while the red dotted line indicates biosensor repression at 0 h. G40, glucose 40 g L^−1^; NS, no carbon source added; G1, glucose 1 g L^−1^; X50, xylose 50 g L^−1^. All cultivations were performed in independent duplicate experiments.

Finally, we overexpressed *SNF3* under a constitutive promoter to test whether signal enhancement would occur, in contrast to the reduced *HXT2p* fluorescence observed after *SNF3* deletion. One copy of the *SNF3* overexpression cassette was introduced into biosensor strains either lacking or harboring the recombinant XR-XDH xylose metabolic pathway, resulting in TMBB33 (TMB3713; *SNF3* OE) and TMBB34 (TMB3753; *SNF3* OE) strains, respectively (Table 1). The corresponding results are presented in Fig. 4, with supporting flow cytometry data summarized in Table S6. Overexpression of *SNF3* led to higher baseline *HXT2*p activity—i.e. activation of Snf3p pathway in the absence of any ligand (sugar)—for both strains cultivated in YNB-only medium (Fig. 4). Under this condition, an induced peak of *HXT2*p fluorescence was detected compared to 0 h (Fig. 4B and 4D; Table S6). In the strain unable to metabolize xylose, exposure to low glucose strongly elevated *HXT2*p induction when *SNF3* was overexpressed (TMBB33; Fig. 4A), with approximately 50% higher induction compared to the *SNF3* wild-type strain (TMB3713). In contrast, the effect of xylose was less marked: although a significant increase in *HXT2*p activity above the basal level was noticed, this only occurred after 12 h of cultivation (Fig. 4A and 4B). These observations suggest that xylose on its own acts as a weak and non-specific ligand for Snf3p, probably mimicking glucose in structure. In contrast, when xylose metabolism is present alongside *SNF3* overexpression, maximal *HXT2*p induction is achieved (TMBB34; Fig. 4C) on xylose, reaching levels comparable to those observed under low-glucose. These results further cement that intracellular metabolites from xylose catabolism help to complete *HXT2*p derepression alongside Snf3p activation.

In summary, Snf3p can act as a weak sensor of xylose, with signals from metabolic intermediates amplifying the response, leading to elevated *HXT2* de-repression and induction.

### Can xylose sensing be increased with the help of a Snf3-based chimeric receptor?

Activation of the Snf3p/Rgt2p pathway on xylose may be beneficial to utilization rates as it leads to the expression of promiscuous transporters capable of pentose uptake. To explore this, we attempted to engineer the specificity of the Snf3p/Rgt2p pathway for robust and complete activation on xylose. To increase the xylose signaling, xylose-specific chimeric receptors were constructed by attaching the C-terminal tail (signaling domain) of the low glucose sensor Snf3p to the transmembrane domains of a xylose-binding Gal2pmut transporter protein (Fig. S3). The chimeric sensor approach was designed based on previous findings showing that fusing the Snf3p receptor signaling domain (“tail”; residues from 543 to 884) to the transmembrane domains of Hxt1p and Hxt2p hexose transporters partially re-established the glucose-induced signals when the native sensors were deleted (Ozcan et al. 1998, Kim et al. 2024). Therefore, we aimed to replace the glucose-binding domain of Snf3p with the xylose-binding domain of a Gal2pmut transporter protein, which showed 65% sequence identity to the previously tested Hxt1p-derived sugar binding domain. The version of Gal2p with two residues substitutions, N376Y and M435I, that has recently been shown to be superior in xylose specificity (Rojas et al. 2021a), was used in the chimeric construct (Fig. S3). The expression of the chimeric receptor was driven by the native *SNF3* promoter. The choice to use native rather than constitutive promoters was based on the evidence that overexpression of the *HXT1*-*SNF3* chimera led to a constitutive activation of the pathway independent of glucose levels, probably due to receptor overload of the membrane (Ozcan et al. 1998). Lastly, to prevent the ability of the chimeric protein to transport xylose, the C-terminal domain of Gal2p was truncated (residues from 531 to end; Fig. S3) since this had previously been shown to diminish sugar transport in Hxt1p (Scharff-Poulsen et al. 2018).

**Figure 5.**
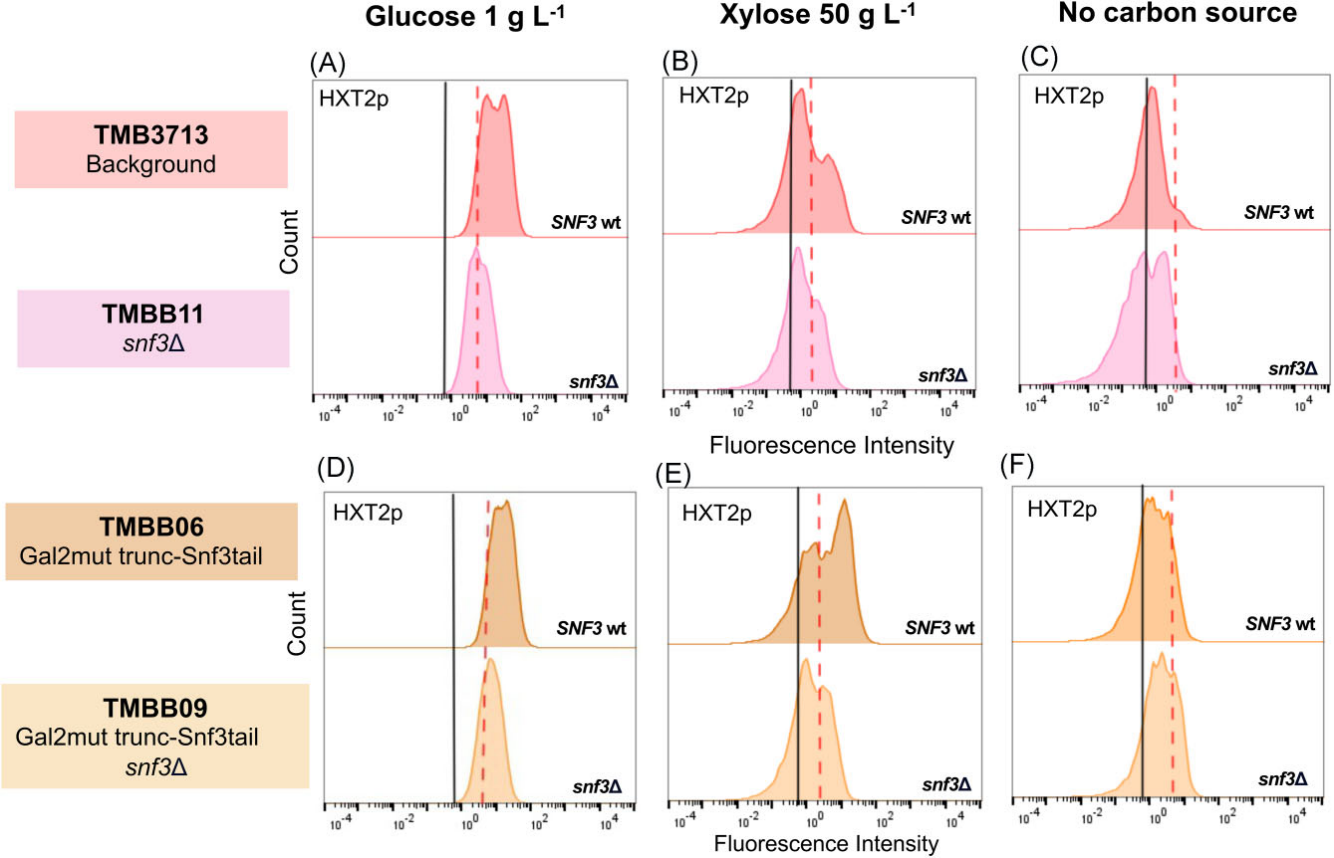
**Effect of a chimeric xylose transporter/receptor on *HXT2*p-GFP signals in *SNF3* wild-type and *snf3*Δ strains**. Expression of the chimeric receptor in TMBB06 moderately enhanced the induction signal under the xylose condition **(E)** compared to TMB3713 **(B)**. However, the chimeric receptor alone was unable to restore the signal in the TMBB09 strain lacking SNF3 (E; lower panel). The conditions tested were YNB media with low glucose (1 g L^−1^; the biosensor induction condition), high xylose (50 g L^−1^) and the medium without any sugar supplementation. Experiments were performed in biological duplicates in shake flasks, representative histograms are shown.

The xylose signaling ability of the chimeric receptor was evaluated by determining if its expression caused an increase in the induction of the *HXT2*p-GFP biosensor in the background strain (Table 1). The flow cytometry data in Fig. 5 show that inclusion of the chimeric protein slightly increased the *HXT2*p biosensor FI of the induced population on xylose (Fig. 5E; right peak) compared to the background strain. However, the response was not significantly different from that obtained by including only the Gal2pmut transporter itself (TMB3723 strain in Fig. 2E). These results could indicate that the chimeric construct acted to induce *HXT2*p-GFP via the xylose transport previously described above rather than via xylose-specific signaling.

**Figure 6.**
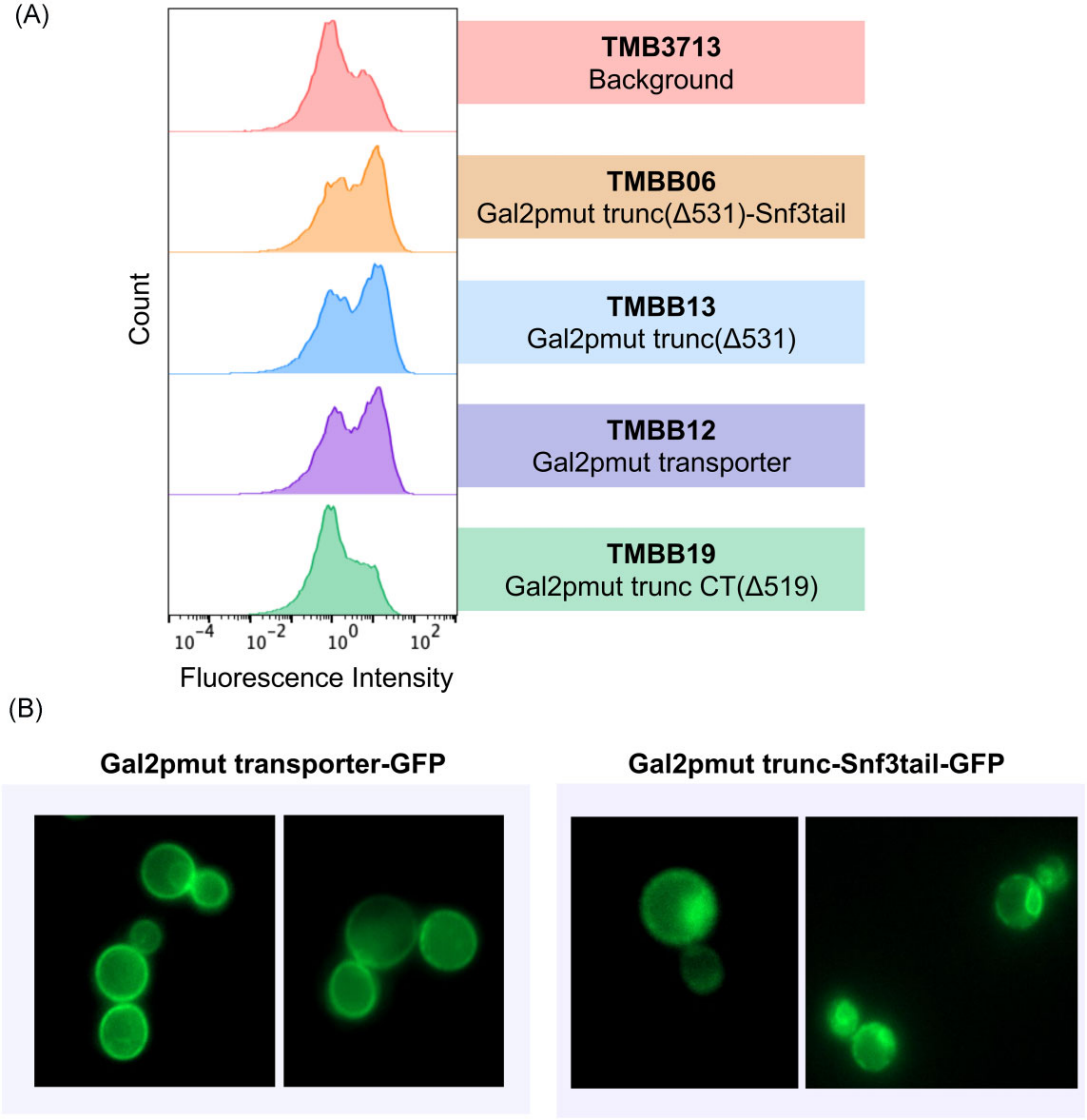
**Fluorescence profiles of the *HXT2*p biosensor for mutated Gal2p constructs (A) and subcellular localization of the Gal2p transporter and the chimera tagged with GFP (B)**. The different variants were derived from the Gal2p xylose transporter containing the two substitutions N376F and M435I (Gal2pmut; Rojas et al. 2021a). In **(A)**, strains with the following modifications were cultivated on xylose 50 g L^−1^: addition of chimera (TMBB06; truncated Gal2p mut transporter with Snf3 tail); addition of truncated Gal2p without Snf3p tail (TMBB13); addition of entirely deleted C-terminal Gal2pmut (TMBB19); and addition of Gal2pmut transporter (TMBB12). Experiments were performed in biological duplicates in shake flasks, representative histograms are shown. **(B)** shows the localization of the Gal2pmut transporter (left panel) and the chimeric Gal2pmut transporter-sensor fusion (right panel). These constructs were expressed as C-terminal GFP fusions from multicopy plasmids and visualized using fluorescence microscopy. Gal2pmut localizes stably to the plasma membrane, whereas the chimera displays only partial membrane localization, with internal signal suggestive of intracellular retention or mislocalization.

To investigate this possibility, the Gal2pmut transporter was truncated in the same manner and integrated in the background strain (resulting in TMBB13 strain). Addition of this variant of the protein still resulted in the same fluorescent signal as seen for both the chimeric protein and the non-truncated Gal2p, indicating that the truncation was likely insufficient to stop sugar transport (Fig. 6A). To address this, a more drastically truncated variant of the mutated Gal2p transporter was created (from residues 519 to end; TMBB19 strain), this time missing a more substantial part of the C-terminus. With this variant, the fluorescent response reverted to the one of the background strain (Fig. 6A), indicating that transport was successfully hindered. Further investigation revealed that the complete C-terminal truncation had previously been described to abolish sugar transport presumably due to a lack of localization of the transporter to the membrane (Rojas et al. 2021b). As such, the addition of the *SNF3* cytoplasmic tail likely just led to re-localization of the chimeric protein to the membrane and thus resumed transport. To investigate this, the subcellular localization of the non-truncated Gal2pmut transporter and the chimeric receptor was assayed using GFP-tagged constructs and fluorescence microscopy (Fig. 6B). The non-truncated Gal2pmut transporter was observed on the plasma membrane, while the chimera appeared only partially membrane localized. Thus, the chimera may be less stable than the original transporter, showing intracellular fluorescence suggestive of mislocalization and/or degradation (Fig. 6B). The reason why the Gal2pmut transporter and the chimera produced similar *HXT2*p induction signals despite their clear differences in localization and apparent stability is not yet clear. It is likely that expressing these constructs results in a generally low threshold for *HXT2*p activation in the TMB3713 background strain. Furthermore, the findings suggest that the observed signals are neither entirely dependent on nor clearly magnified by the Snf3p tail fusion, despite our original hypothesis that its attachment to the transporter would enhance signaling. Thus, further studies will be required to elucidate the precise mechanisms involved. Future attempts might benefit from integrating the chimeric protein into a background lacking sugar transporters but capable of xylose utilization to quantify the transporting capabilities of the chimera and enable further troubleshooting. Additionally, since correct membrane assembly and stability are critical for sensor function, future chimeras may be favored by optimizing membrane targeting or linker regions.There was still a possibility that the chimeric protein was functional despite also transporting xylose, by functioning as a transceptor. Transceptors have been described in *S. cerevisiae* and other organisms as nutrient transporters that also function as receptors (Holsbeeks et al. 2004, Gojon et al. 2011, Schothorst et al. 2013). In the results above, we showed that deletion of *SNF3* abolished the effect of the Gal2p transporter gene overexpression (Fig 2E). Thus, any contribution to the fluorescence by xylose transport from the chimeric receptor should be eliminated in a strain lacking *SNF3*, allowing us to highlight any effects from xylose-specific sensing (Fig. 5E; lower panel). However, deletion of the wild-type Snf3p receptor gene and addition of the chimeric one did not maintain the induction signal, and expression of the *HXT2*p biosensor was reduced by more than 50% (Table S3; refer to strains TMBB06 and TMBB09). This indicates that the chimera alone failed to restore the signals from both extracellular xylose and low glucose, and ultimately suggests that the chimeric truncated Gal2mut-Snf3tail construct was unable to bind xylose to productively transduce a signal.

As a last attempt to build a working chimeric sensor, we also constructed a Rgt2-based hybrid protein following a similar approach. Although this Gal2mut-Rgt2tail chimera showed initial promise with xylose-specific responses (data not shown), we were unable to conclusively show that the response from the Rgt2-based chimera was caused by binding of xylose to the receptor despite the use of extensive controls. Ultimately it was abandoned due to the complexity of other signaling pathways acting on the expression of genes responsive to the Rgt2 receptor. Induction of the *HXT1*p-GFP biosensor depends not only on the signals transmitted by the high glucose receptor Rgt2p, but also on signals from the PKA pathway via hyperphosphorylation of Rgt1p, the SNF1 pathway via Mig1p and Hxk2p and complementary signals from Hog1p (Brink et al. 2021). As such, we chose instead to further focus on investigating the effects of *SNF3* deletion and overexpression on culture performance and the putative detrimental effects of Snf3p-based xylose sensing.

### Is the impact of Snf3p activation by extracellular xylose physiologically relevant?

**Figure 7.**
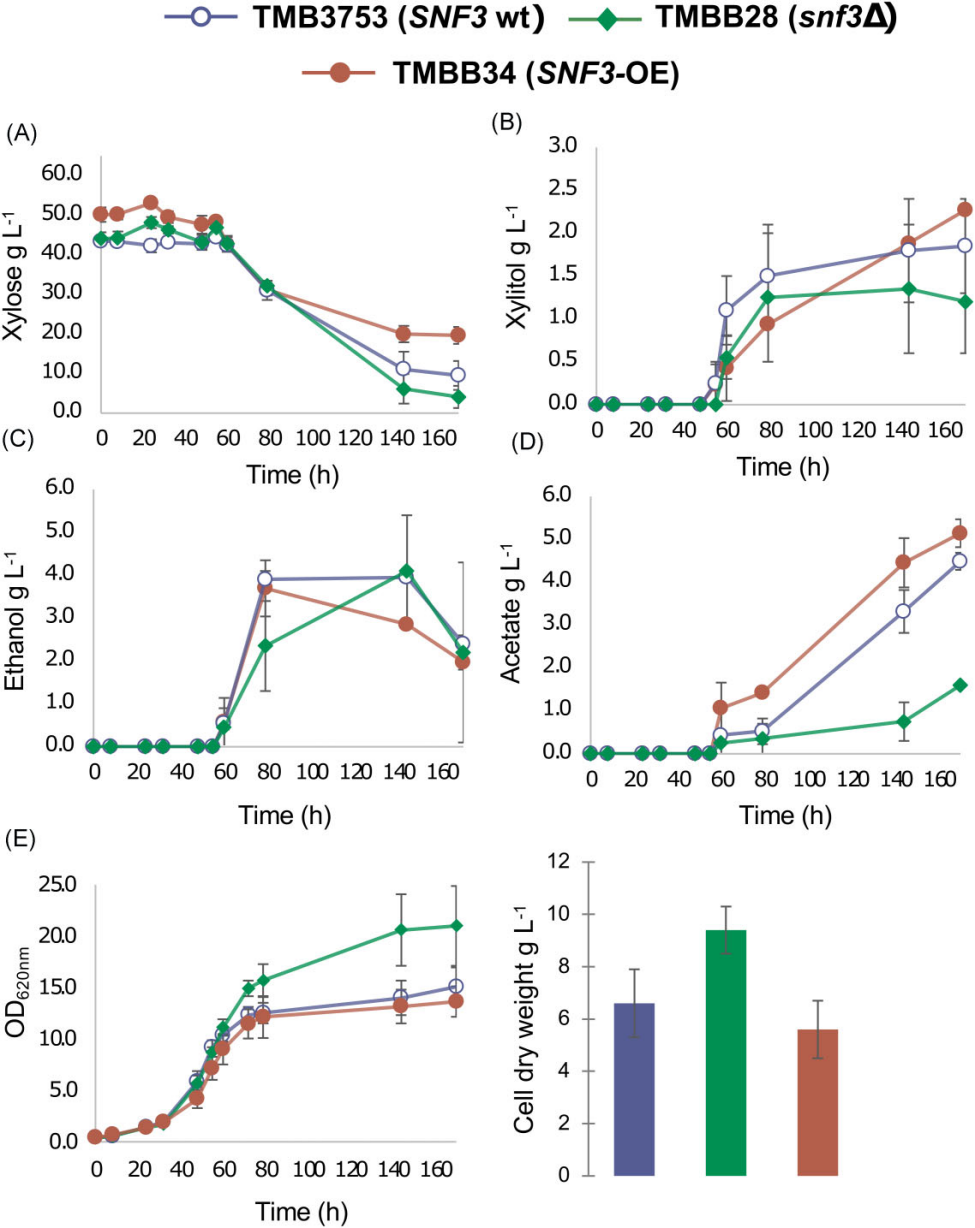
**Cultivation profiles on xylose for the engineered xylose-assimilating strain TMB3753 and its counterparts, TMBB28 (*SNF3-*deleted) and TMBB34 (*SNF3* overexpressing)**. Xylose consumption, product accumulation, and biomass growth were assessed in YNB media containing 50 g L^−1^ xylose in baffled flasks (A–E). Data shows the average and standard deviation of two biological duplicates.

As the sensing of xylose by Snf3p could have both beneficial (increased transport) and detrimental (increased maintenance cost) effects on xylose utilization, we performed a comparative cultivation of the three strains carrying the XR/XDH pathway: *SNF3* wild-type (TMB3753), *SNF3* deletion (TMBB28), and *SNF3* overexpression (TMBB34). The experiments were performed in shake flasks containing 50 g L^−1^ xylose as the only carbon source. The growth characteristics, xylose consumption, and byproduct accumulation were determined for the three strains (Fig. 7). Based on cell dry weight values, the *SNF3*- deletant exhibited increased final biomass compared to both the wild-type and *SNF3*-overexpressing strains (Fig. 7E), in addition to a slightly higher specific growth rate (Table S7). Changes in biomass production between the *SNF3* deletion and *SNF3* overexpression strains were accompanied by shifts in metabolite accumulation profiles (Fig. 7B-D; Table S7). The most noticeable effect was on acetate production, which was reduced by more than 50% in the absence of *SNF3* (Fig. 7D). In contrast, overexpression of *SNF3* (TMBB34) increased byproduct yields compared to the deletant strain (TMBB28), at the same time that xylose consumption was impaired (Table S7). In TMBB34, impairment of xylose utilization and biomass accumulation implies that overexpressing *SNF3* may impose a metabolic burden, likely arising from the energy and resource allocation to maintaining high levels of the sensor, reducing energy available for biomass synthesis and sugar transport. Consequently, cells may redirect carbon from biomass formation toward byproduct synthesis. In fact, even though we showed in the previous section that *SNF3* overexpression boosted *HXT2* induction (TMBB34 results; Fig. 4C), this does not appear to effectively contribute to xylose uptake, probably because the transport capacity of Hxt2p is too low to support adequate internalization under high-xylose conditions. When comparing *SNF3*-deletant (TMBB28) and *SNF3* wild-type (TMB3753) performance, xylose uptake rate was not substantially affected by the presence or absence of *SNF3* (Fig. 7A). This again suggests that *HXT2* induction via Snf3p signaling in the native background does not significantly influence sugar consumption when the extracellular levels of xylose are high. As such, the constitutively expressed Gal2p-N376F transporter likely dominates xylose transport in all the strains.

## Discussion

In this study, we showed that xylose triggers the induction of the glucose-scavenging transporter gene *HXT2* in a *SNF3*-dependent manner. This was accomplished using a transcription-based *HXT2*p-GFP biosensor in *snf3*-null and *SNF3-*overexpressing strains of *S. cerevisiae* equipped with partial or full xylose metabolism, enabling the identification of which genetic components contribute to the activation of the signaling pathway. By characterizing the xylose-based Snf3p activation in the absence of glucose and in a strain background which carries wildtype sugar signaling pathways (Vanhalewyn et al. 1999), prior hypotheses of the involvement of the Snf3p receptor in *HXT2* induction on xylose (Wu et al. 2020) could be directly confirmed and be shown to be relevant to the wildtype. The induction of *HXT2* by Snf3p is usually only seen in conditions where glucose levels are low (Brink et al. 2021), and our results are consistent with previous reports of the non-fermentative, low glucose-like response on xylose (Matsushika et al. 2014, Osiro et al. 2018).

Snf3p was previously shown by Dietvorst et al. (2010) to sense other sugars such as fructose and mannose, as well as glucose analogs, in addition to glucose itself. However, the authors reported that this was not the case for xylose as no Mth1p co-repressor degradation was detected. Although this seems to contradict our results, measuring Mth1p degradation as a proxy for Snf3p signaling may not be a sufficiently sensitive method for detecting the subtle effects that xylose exerts on this pathway—especially not with the presence of subpopulations which may skew interpretations. Our results are also supported by the majority of transcriptome studies on xylose cultures which show high expression levels of both *SNF3* and *HXT2* (Jin et al. 2004, Salusjärvi et al. 2008, Matsushika et al. 2014, Zeng et al. 2016, Wu et al. 2020). This is in opposition of the canonical response observed during complete glucose depletion, where *HXT2* expression is repressed (Brink et al. 2021). Additionally, while the high glucose sensor Rgt2p is rapidly degraded in glucose scarce media, the Snf3p low glucose sensor remains stable at low and depleted concentrations (Kim and Rodriguez 2021, Kim et al. 2024). These reports alone indicate that Snf3p is present under xylose conditions and that *HXT2* may be responsive to xylose, which our results corroborate by showing a direct link between *SNF3* and *HXT2* expression in the presence of xylose.

Moderate-to-high affinity glucose transporters Hxt2p and Hxt4p—controlled by the Snf3p signaling pathway—have the capacity to transport xylose (Hamacher et al. 2002, Sedlak et al. 2004, Gonçalves et al. 2014). As such, the importance of xylose sensing by Snf3p could then be linked to the need to express transporters able to take up this pentose sugar. However, the underlying physiological purpose and significance remains undetermined as *S. cerevisiae* does not naturally ferment xylose. It is possible that the redox factors generated by XR during conversion to xylitol may be beneficial enough to warrant upregulating xylose internalization. It may also be an evolutionary remnant from a past time where *S. cerevisiae* did ferment xylose. Adaptive laboratory evolution of a few *S. cerevisiae* strains did lead to growth on xylose without the need for recombinant technology (Attfield and Bell 2006), indicating that key genetic components may be present in some specific strains. Sensing may also result from substrate promiscuity. The Snf3p receptor shares high similarity with hexose transporters and has been suggested to derive from one such protein that lost the ability to transport sugars (Donzella et al. 2023). Given that these transporters are known to be promiscuous (Hamacher et al. 2002, Sedlak et al. 2004, Gonçalves et al. 2014), it is not unthinkable that the sensors might have the same ability. Future studies are needed to elucidate the purpose. One might look at homologous sugar-sensing proteins in efficient native xylose-utilizers such as *Spathaspora passalidarum* to uncover whether similar or divergent signaling mechanisms regulate transporter induction and metabolic capacities in these organisms.

By employing strains with varying levels of xylose metabolizing capacity, we were able to investigate the effects of *SNF3* deletion in three different contexts with xylose being either: (i) extracellular, (ii) extracellular and intracellular, or (iii) extracellular and intracellularly metabolized. While the overall effect of deleting *SNF3* was a reduction in *HXT2* induction regardless of the metabolic capacity of the cell, the deletion appeared to have a more definitive impact in non-metabolizing strains. Without the addition of the xylose utilization pathway, the strain displayed prominent subpopulations of induced and repressed cells and the deletion of *SNF3* prompted the disappearance of the induced population, regardless of the internalization of xylose or not. Upon inclusion of the xylose metabolic pathway, however, the repressed subpopulation was abolished entirely and deletion of *SNF3* no longer caused the induced population to disappear. This indicates that, although xylose triggers *HXT2* induction via Snf3p, another mechanism must be responsible for the full induction observed during active xylose utilization. This was in line with the results from overexpressing *SNF3*, which ultimately confirmed that xylose had to be metabolized and Snf3p had to be active in order to maximally induce *HXT2*. One possible explanation is that metabolized xylose-derived intermediates contribute to *HXT2*p expression through crosstalk with other signaling routes, such as SNF1/Mig1p (Fig. 1). Activation of the SNF1 complex leads to phosphorylation of the Mig1p repressor, which in turn leads to the dissociation of the Cyc8p-Tup1p co-repressor complex and derepression of *HXT2* (Brink et al. 2021). Supporting this hypothesis, Osiro et al. (2018) showed that high xylose levels effectively activate the SNF1/Mig1p pathway-responsive *SUC2*p biosensor in the TMB3753 strain. This led to the conclusion that xylose acts as if it were low glucose, with both *SUC2*p and *HXT2*p biosensors consistently responsive to xylose. Unlike the extracellular sensing function of Snf3p, the SNF1/Mig1p pathway is controlled intracellularly (Vincent et al. 2001, Hedbacker 2008) and must therefore rely on xylose import and metabolism. In addition to this, glycolytic metabolites have been demonstrated to act as regulators in signal transduction mechanisms, linking the metabolic status of the cell to the signaling machinery (reviewed in Brink et al. 2021). For example, these intermediates stimulate the PKA pathway and can also contribute to gene repression, as observed for *SUC2*p (Borgström et al. 2022). Conversely, xylose seems to have an opposite effect, likely due to differences in the accumulation profiles and concentrations of glycolytic intermediates between xylose and glucose metabolism (Borgström et al. 2022). However, derepression of *HXT2* via the SNF1/Mig1p pathway does not satisfactorily explain why the Rgt1p-Cyc8p-Tup1p complex is not immediately reformed without Snf3p triggering Std1p degradation. Further in-depth studies on the detailed mechanisms of the Snf3p/Rgt2p pathway are needed to understand this crosstalk, including elucidating the mechanism for recruitment of Std1p to the membrane and mechanism for nuclear degradation of Mth1p under low glucose conditions. Overall, this perspective needs to be expanded upon to link xylose intermediates to *HXT2*p induction signals in the xylose-utilizing strain.

The observation that the extracellular xylose signal in *S. cerevisiae* is limited and relying on the glucose sensing machinery led us to postulate that glucose sensors could be engineered to specifically detect xylose, with the long-term goal of improving xylose utilization. To this end, we pursued a chimeric receptor engineering strategy by coupling a truncated version of the xylose-specific Gal2pmut transporter to the regulatory cytoplasmic tails of the Snf3p protein. Unfortunately, no restoration of the *HXT2*p induction signal was achieved. Two distinct but interrelated structural reasons may explain this outcome. First, although hexose transporters such as Gal2p and glucose sensors share a high degree of structural similarity, the amino acid sequence is quite dissimilar. Certain amino acids located in the transmembrane domains of Snf3p have been indicated to likely be required for extracellular recognition of the sugar and complete activation of the signaling cascade (Ozcan et al. 1998). In addition to identifying these residues, mutations prohibiting sugar uptake without affecting the localization would also need to be identified to engineer a fully functional chimeric receptor. Second, recent research by Kim et al. (2024) demonstrated that chimeric constructs between glucose transporters and signaling tails only partially result in sugar signaling, likely because Yck kinases—key components of the Snf3p/Rgt2p cascade—do not properly phosphorylate the tail domains of Hxt1p chimeras. This may lead to unstable configurations of the hybrid hexose transporter/sensor proteins and degradation (Kim et al. 2024).

In our study, the metabolic characterization of *SNF3* gene deletion on xylose revealed an unexpected phenotype, as increased final cell density was recorded. Thus, xylose activation of this receptor seems to be detrimental to the cell rather than beneficial, the opposite of what we could hypothesize. Although the Hxt2p and Hxt4p transporters have been observed to promiscuously transport xylose (Hamacher et al. 2002, Sedlak et al. 2004, Gonçalves et al. 2014), it is possible that the gain in transport rate is outweighed by the above-mentioned drawbacks of producing the transporters. The increase in final cell density may reflect a decrease in the required cellular maintenance energy, for instance by eliminating the unnecessary transporter production. This is supported by the fact that xylose utilization remained unchanged despite the increased biomass formation. An inverse phenotype occurred upon *SNF3* overexpression; however, the lower biomass and xylose utilization could instead be related to energy drainage from constitutive gene expression. It should be noticed that xylose-metabolizing strains in this study express a dedicated xylose transporter (Gal2p mutant), which may alleviate internalization as a bottleneck and with it any benefits derived from Hxt2p/4p expression. Future studies should be conducted in strains lacking a xylose-specific transporter and under low xylose levels, as the transporters induced by the Snf3p pathway are considered low capacity and may only contribute significantly to sugar uptake under such limiting conditions. This would allow a better understanding of the relationships between Snf3p activation signals and the native transporter expression for xylose uptake. Overall, changes in *SNF3* levels might have the potential to improve bioproduct yields from xylose, especially in strains already carrying a xylose-specific transporter. However, additional experiments should be performed, in particular under anaerobic conditions to assess whether this modification is applicable to the current second-generation ethanol industry.

Until now, it has been unclear whether *S. cerevisiae* senses the presence of extracellular xylose. Our findings demonstrate that—in addition to low glucose—Snf3p receptor also responds to high extracellular xylose, which probably contributes to the peculiar regulatory response of *S. cerevisiae* on xylose. It remains unclear if xylose is an intentional binding partner to Snf3p, or if this is a result of promiscuous binding. Snf3p activation by xylose rather than glucose had previously been reported to result in a weaker induction of *HXT2* based on qPCR assays, indicating that the affinity of the receptor to xylose might be lower or that xylose is not capable of fully activating the signaling pathway (Wu et al. 2020). With the use of flow cytometry, we demonstrated that the population-wide decrease in receptor activity is due to individual cells not responding to xylose rather than a partial activation of each cell. Additionally, with a functional xylose assimilation pathway, intracellular signals cooperate to fully induce *HXT2*. Construction of a xylose-specific chimeric receptor based on Snf3 sensing tail and Gal2pmut xylose affinity may improve the xylose-specific sensing; however, the current trials were unsuccessful. After deletion and upregulation of Snf3p membrane sensor gene, changes in biomass production and byproducts accumulation were observed, indicating direct impact of the signaling pathway on xylose catabolism. Still, a more complete view of how xylose sensing influences xylose fermentation in recombinant *S. cerevisiae* strains is needed. Further studies should focus on other regulatory components in the sensing machinery of *S. cerevisiae* that may ultimately be targeted to promote an overall efficient fermentation response, as in the case of glucose, for efficient valorization of lignocellulosic biomass.

## Supplementary Material

foaf040_Supplemental_File
