# Supplementary material for: Glucose receptor deletion and engineering: impact on xylose sensing and utilization in Saccharomyces cerevisiae
Source: FEMS Yeast Res. 2025 Jul 29;25:foaf040. doi: 10.1093/femsyr/foaf040 (PMC12359139; doi:10.1093/femsyr/foaf040)
Supplement: foaf040_Supplemental_File [file foaf040_supplemental_file.docx]

**Supporting information for:**

***SNF3* receptor deletion and engineering: impact on xylose sensing and utilization in *Saccharomyces cerevisiae***

Bruna C. Bolzico^1,2†^, Viktor C. Persson^3†^, Raul N. Comelli^1,2^, Marie Gorwa-Grauslund^3^

**^1^**Grupo de Procesos Biológicos en Ingeniería Ambiental, Facultad de Ingeniería y Ciencias Hídricas, Universidad Nacional del Litoral, Santa Fe, Argentina

^2^Consejo Nacional de Investigaciones Científicas y Técnicas (CONICET), Argentina

^3^Biotechnology and Applied Microbiology, Department of Process and Life Science Engineering, Lund University, Lund, Sweden

*Corresponding author. Email address: [biotech.viktor@outlook.com](mailto:biotech.viktor@outlook.com)

^†^Bruna C. Bolzico and Viktor C. Persson contributed equally to this work

Tables and Figures related to Materials and methods section:

- Supplementary Table 1: List of plasmids
- Supplementary Table 2: List of primers
- Supplementary Figure 1: Isolation of subpopulations

Tables and Figures related to Results section:

- Supplementary Figure 2: Autofluorescence control strains histograms
- Supplementary Figure 3: Schematic structure of transporter and chimeric sensor
- Supplementary Table 3: Flow cytometry parameters for *SNF3* deletant strains
- Supplementary Table 4: Flow cytometry parameters for *SNF3* deletant strains
- Supplementary Table 5: Normalized flow cytometry data for *SNF3* deletant strains
- Supplementary Table 6: Flow cytometry parameters for *SNF3* overexpressing strains
- Supplementary Table 7: Growth and metabolites accumulation parameters

**Table S1.** List of plasmids used and constructed in this work.

| **Plasmid designation** | **Relevant characteristics** | **References** |
| --- | --- | --- |
| **pCfB3036** | XI-1-Markerfree backbone | Jessop-fabre et al. (2016) |
| **pCfB3042** | *SNR52*p-gRNA_X4-CYC1t; natMX | Jessop-fabre et al. (2016) |
| **pCfB2312** | *TEF1*p-*Cas9*-*CYC1*t; kanMX | Jessop-fabre et al. (2016) |
| **pUC57+ ClosXI** | pUC57; TEF1p- LpXImut2 -GPM1t | Persson et al. (2023) |
| **pRS42N** | TEF1p-natMX-ADH1t | Taxis (2018) |
| **YIpGFP** | yEGFP3-PGK1t | Brink et al. (2016) |
| **pBB01** | pUC57*;SNF3*p-*GAL2*^N376Y/M435I^trunc-*SNF3*tail- *SNF3t* | This study |
| **pBB01INT** | pCfB3036; *SNF3*p-*GAL2*^N376Y/M435I^trunc-*SNF3*tail-*SNF3*t | This study |
| **pBB03INT** | pBB01INT*; SNF3*p-*GAL2*^N376Y/M435I^-*SNF3*t | This study |
| **pBB04INT** | pBB01INT*; SNF3*p-*GAL2*^N376Y/M435I^trunc-*SNF3*t | This study |
| **pBB05INT** | pBB01INT*; SNF3*p-*GAL2*^N376Y/M435I^trunc CT-*SNF3*t | This study |
| **pBB07** | pRS42N; *SNF3*p-*GAL2*^N376Y/M435I^trunc-*SNF3*tail-GFP- *SNF3t* | This study |
| **pBB08** | pRS42N; *SNF3*p-*GAL2*^N376Y/M435I^-GFP-*SNF3*t | This study |
| **pBB09** | pBB07; *TDH3*p-*GAL2*^N376Y/M435I^trunc-*SNF3*tail-GFP- *SNF3t* | This study |
| **pBB10** | pRS42N; *TDH3*p-*GAL2*^N376Y/M435I^-GFP-*SNF3*t | This study |
| **pBB11** | pUC57; TEF1p-SNF3-GPM1t | This study |
| **pBBg5** | *SNR52*p-gRNA_*SNF3*-*CYC1*t; natMX | This study |
| **pBBg12** | *SNR52*p-gRNA_XI-1-*CYC1*t; natMX | This study |

**Table S2.** List of primers used in the current work.

| Primer name | Sequence (5'-3') | Description |
| --- | --- | --- |
| BBg6_Rv | GATCATTTATCTTTCACTGC | General reverse primer for gRNA construction |
| BBg5_Fw | GAAACGACGCATCTTCGTATGTTTTAGGCTAGAAATAGCAAG | gRNA plasmid construction. 20 pb targeting *SNF3* |
| BBg12_F | GCGGTGCACGGATTTCAGCAGTTTTAGAGCTAGAAATAGCAAG | gRNA plasmid construction  20 pb targeting XI-1 locus |
| LW_8 | gctatactgctgtcgattcg | gRNA plasmid sequencing |
|  |  |  |
| BB01_Fw | GTCAATTATTCTATAATCTGGGGAAATTCAGTCATACTGAGAAAAACTAGGAATGAAGCCATACCAAACG | DNA donor amplification. 50pb tail for *SNF3* deletion |
| BB02_Rv | AATTATAATGCACGTCCGCTTAATTAATACATCGAATAACATTAAATTAATCAGCGATCTGTCTATTTCG | DNA donor amplification. 50pb tail for *SNF3* deletion |
| BB03_Fw | AGCCTTCTCAACCGCCAAC | Verification of *SNF3* deletion |
| BB15_Rv | CTACAGGCATCGTGGTGTCA | Verification of *SNF3* deletion |
| BB06_Fw | GGGTCGCATACATCATCATTAGG | Verification of internal *SNF3* |
| BB07_Rv | GTCAGCCATCACGGAGAGAG | Verification of internal *SNF3* |
| BB56_Fw | agattaGCGATCGCTAAATGGATCCTAATAGTAACAGTTC | SNF3 amplification adding SfAI site |
| BB57_Rv | agattaCGCCGGCGTTATTTCAAATCATTATTTTCATTTACAGG | SNF3 amplification adding MreI site |
| BB04_Rv | CATTACGCCAAGAGAAACAGGG | SNF3 verification |
| HR-TEF1p_X-4_US_Rv | tccgagaaaatctggaagagtaaaaaaggagtagaaacattttgaagctatCTGCTCTTGAATGGCGACAG | Amplification of targeting X-4 fragment, adding 56 pb homology to the TEF1 promoter |
| X-4_US_Fw | CCCAAAGCTAAGAGTCCCATTTTATTC | Amplification of targeting X-4 fragment |
| HR-PGM1t_X-4_DS_Fw | GTAGGAGTGCACCAATTGCAAAGGGAAAAGCTGAATGGGCAGTTCGAATAACAGGCATGGGAAGATTCG | Amplification of targeting X-4 fragment, adding 55 pb homology to GPM1 terminator |
| X-4_DS_Rv | CTGGTGAGGATTTACGGTATG | Amplification of targeting X-4 fragment |
| X-4_Ver_Int_Rv | CAGAGATTGATAGGAAGGGTGG | Verification of expression cassette integration in X-4 site |
| 35-36_Fw | actgggaaaaccctggcg | Amplification of pCfB3036 integrative backbones |
| 36_Rv | aggttaGGCGCGCCctccgagaaagtttgatgcg | Amplification of pCfB3036 backbone, adding AscI site |
| XI-1_Ver_Cloning_R | ccccaactagcaaccctc | Verification of pBB01INT  Construction |
| Int_SNF3_tail_Ver_Fw | CACTCAAGCGAAGAAAACGC | Verification of pBB01INT construction and integration of Snf3 chimera |
| XI-1_Ver_Int_Rv | CTTGTGTTGATGCCAACGAC | Verification of expression cassette integration in XI-1 site |
| BB51_F | gcagtatgtatcatcgcc | Sequencing of pBB01INT  integrative plasmid |
| BB52_R | GACTACACCAATGACAATGG | Sequencing of pBB01INT  integrative plasmid |
| BB53_F | ACTGGCTGGCAATGCG | Sequencing of pBB01INT  Integrative plasmids |
| GAL2_NcoI_Rv | agattaCCATGGcgagttattctagcatggccttg | Amplification of *GAL2* adding NcoI site |
| GAL2_MfeI_Fw | cacagcagaatcattcccac | Amplification of *GAL2* |
| GAL2_BglII_Fw | CCATTGCTAAGTCTAACAAGGTGTC | Amplification of *GAL2* |
| GAL2trunc_NcoI_Rv | agattaCCATGGTTAAATTTCTTCTAACGATAGGCCTTTAGTTTCTG | Amplification of *GAL2* truncated adding NcoI site |
| GAL2trunc_full_NcoI_Rv | agattaCCATGGTTAAAAGAAAAAGACATAAAAAAACATGGCAACCAAAC | Amplification of *GAL2* fully C-terminal truncated adding NcoI site |
| BB55 | CCTGAAGGCTCCCAAAGTG | Sequencing constructs |
| V43_Rv | ttatttgtacaattcatccataccatg | Amplification of GFP ORF |
| V41_Fw | TAGAGGATTTACAACATGACGACAAACCGTGGTACAAGGCCATGCTAGAAatgtctaaaggtgaagaattattcac | Amplification of GFP adding 50 pb homology to GAL2 3’ end |
| V52_Fw | AAAACGCCACTAATCAACCTGTAAATGAAAATAATGATTTGAAACGCGGCatgtctaaaggtgaagaattattcac | Amplification of GFP adding 50 pb homology to SNF3 tail |
| V51_Fw | ACTATAGGGCGAATTGGGTACCGGGCCCCCCCTCGAGGTCGACGGTATCGTTCAGTCTCAAAACTTTCAGTAC | Amplification of SNF3p-GAL2 /GAL2-SNF3tail adding 50 pb homology to pRS42N |
| V50_Rv | gccgcgTTTCAAATCATTATTTTCATTTACAGGTTG | Amplification of SNF3p-GAL2-SNF3tail adding a 6pb linker; without stop codon |
| V47_Rv | ttctagcatggccttgtac | Amplification of SNF3p-GAL2; without stop codon |
| V44_Fw | ttgttactgctgctggtattacccatggtatggatgaattgtacaaataaCCATGGTTAATTTAATGTTATTC | Amplification of SNF3 terminator adding 50 pb homology to the GFP 3’ end |
| V45_Rv | CTAAAGGGAACAAAAGCTGGAGCTCCACCGCGGTGGCGGCCGCTCTAGAAGAGTATCCAAAAGTGTTATAG | Amplification of SNF3 terminator adding 50 pb homology to pRS42N |
| GAL2_BglII_Rv | gacgcattgccagccag | Amplification of the GAL2 5’ end adding BglII site |
| TDH3p_SalI_Fw | agattaGTCGACagtttatcattatcaatactcgcc | Amplification of TDH3p-GAL2 fragment adding SalI site |
| ACT1_Fw | TGGATTCCGGTGATGGTGTT | RT-qPCR reference gene |
| ACT1_Rv | TCAAAATGGCGTGAGGTAGAGA | RT-qPCR reference gene |
| HXT2_Fw | GGCTCTCAACAAACTTCTATCCAC | RT-qPCR target gene |
| HXT2_Rv | GGGAGTTCAGCGTTAGTGTATTC | RT-qPCR target gene |


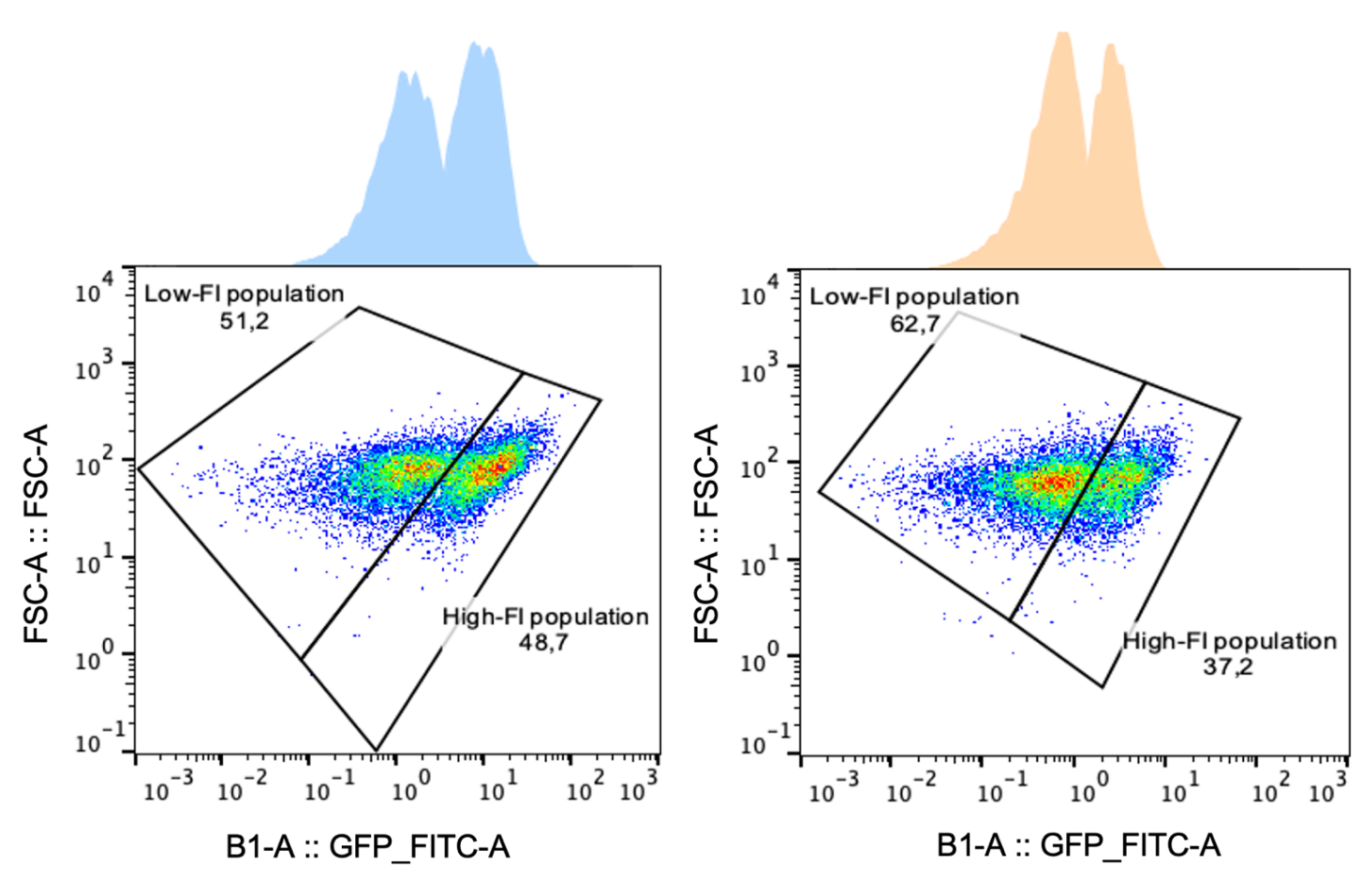


**Figure S1.** Isolation of subpopulations showing distinct fluorescence intensity (FI) during xylose cultivation. The examples illustrate the application of manual gating in the dot plots graphics to isolate the population from the left (low-FI) and the one from the right (high-FI). The values denote the percentage of cells within each gated subpopulation (or cluster) relative to the total number of events evaluated. FlowJo software was employed for the analysis.


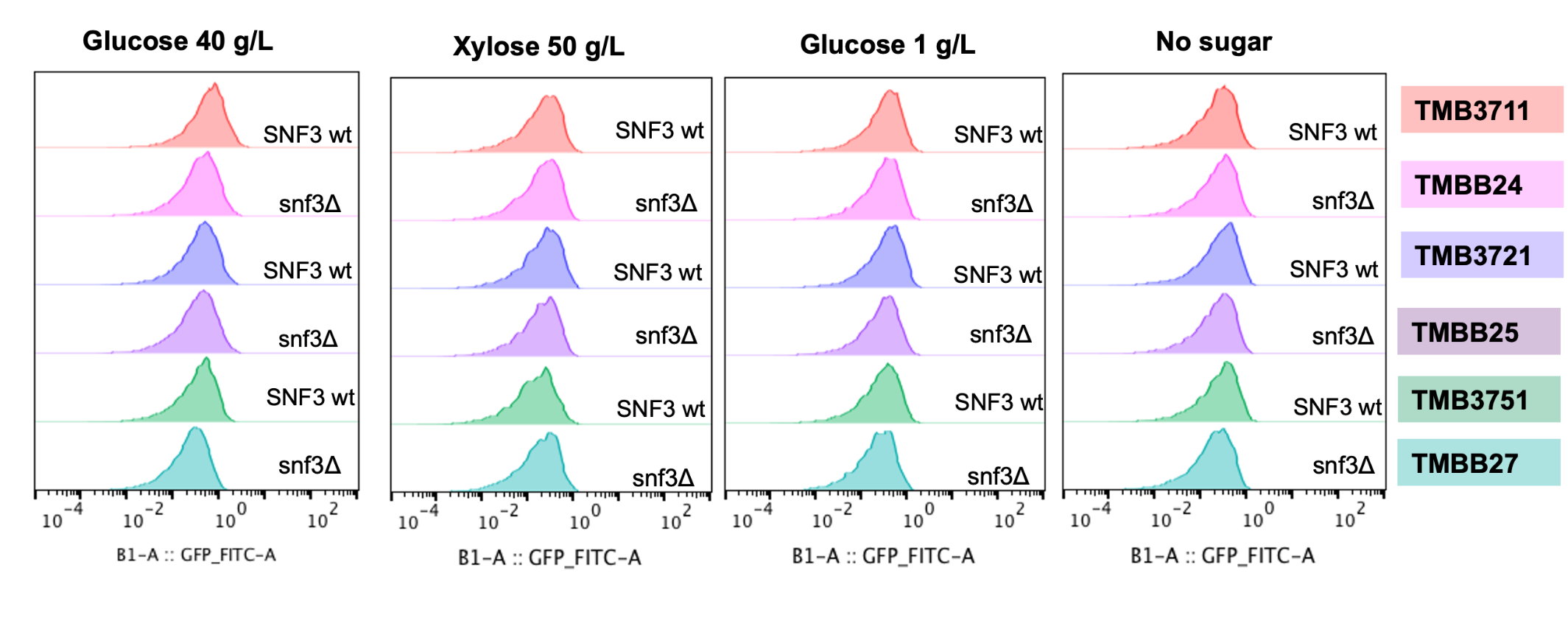


**Figure S2.** Histograms for the autofluorescence control strains (no biosensor) with and without Snf3p under different cultivation conditions after 6 hours. No significant changes in fluorescence intensity profiles or population distribution were observed following *SNF3* knockout in any of the tested conditions. The histograms represent: TMB3711 (background strain) and its corresponding isogenic strain TMBB24 (*SNF3* deleted); TMB3721 (xylose transport) and its isogenic strain TMBB25 (*SNF3* deleted); and TMB3751 (xylose transport + assimilation) and its isogenic strain TMBB27 (*SNF3* deleted). Histograms are representative of biological duplicates from shake flask experiments.

**Table S3.** Signal response of *SNF3* wild-type and *SNF3* mutant strains given by the *HXT2*p biosensor. Values for fluorescence intensity (FI) at 6h and fold change (FC) are presented for different cultivation conditions.

| **Strain** | **Glucose 1 g/L** | | **Xylose 50 g/L** | | | **No carbon source** | |
| --- | --- | --- | --- | --- | --- | --- | --- |
|  | **Mean FI  total population** | **FC** | **Population^*^**  **(%)** | **Mean FI** | **FC** | **Mean FI total population** | **FC** |
| **TMB3713** | 16.05  ±0.49 | 4.11  ±0.35 | High-FI  32 ±3 | 7.11 ±0.74 | 3.36 ±0.93 | 0.82  ±0.49 | 0.31 ±0.06 |
| **TMBB11** | 6.10  ±0.54 | 1.86  ±0.34 | High-FI  29 ±4 | 3.25 ±0.32 | 1.78 ±0.33 | 0.92  ±1.29 | 0.28 ±0.25 |
| **TMB3723** | 13.44  ±0.37 | 4.10  ±0.18 | High-FI  42 ±8 | 10.83  ±2.33 | 3.72 ±0.18 | 2.11  ±0.42 | 0.55 ±0.01 |
| **TMBB26** | 5.43  ±0.52 | 1.82  ±0.05 | High-FI  31 ±2 | 3.33 ±0.33 | 2.16 ±0.32 | 1.07  ±0.13 | 0.59 ±0.09 |
| **TMB3753** | 11.10  ±0.42 | 3.02  ±0.53 | Single | 8.94 ±0.24 | 3.39 ±0.18 | 1.71  ±0.08 | 0.70 ±0.01 |
| **TMBB28** | 5.24  ±0.91 | 1.72  ±0.19 | Single | 6.26 ±0.76 | 2.72 ±0.10 | 2.50  ± 0.11 | 1.12  ± 0.06 |
| **TMBB06** | 19.65  ±1.98 | 4.35  ±0.67 | High-FI  43 ±5 | 14.03 ±3.03 | 4.10 ±0.41 | 1.96  ±0.21 | 0.52 ±0.03 |
| **TMBB09** | 6.54  ±0.17 | 1.78  ±0.02 | High-FI  28 ±10 | 3.32 ±0.30 | 1.61 ±0.34 | 1.46  ±0.58 | 0.50 ±0.04 |

Geometric means of fluorescence histograms were determined in the FITC-A channel at 0h and at 6h for each condition and strain. The FI was normalized to the initial signal to calculate the FC. The experiments were carried out in shake flasks and the values are average of biological duplicates data. **^*^**During xylose cultivation, two subpopulations with different GFP fluorescence were identified for the *HXT2*p biosensor in TMB3713, TMBB11, TMB3723 and TMBB26 strains. The data shown correspond to the peak with high FI or induced. The parameters values for the low-FI and total population are shown in Supplementary Table S4.

**Table S4.**  Flow cytometry parameters of the low-FI and total populations during xylose cultivation and the repression condition (high glucose) for the *HXT2*p biosensor strains with and without *SNF3*.

|  | **Xylose 50 g/L** | | | | | **Glucose 40 g/L** |
| --- | --- | --- | --- | --- | --- | --- |
| **Strain** | **Low-FI Population** | | | **Total population** | | **Total population** |
|  | **Population(%)** | **Mean**  **FI** | **FC** | **Mean**  **FI** | **FC** | **FC** |
| **TMB3713** | 68  ±3 | 0.58  ±0.06 | 0.27  ±0.03 | 1.22  ±0.11 | 0.56  ±0.09 | 1.96  ±0.10 |
| **TMBB11** | 71  ±4 | 0.47  ±0.05 | 0.26  ±0.03 | 1.71  ±0.99 | 0.63  ±0.35 | 1.39  ±0.74 |
| **TMB3723** | 57  ±8 | 1.29  ±0.66 | 0.44  ±0.15 | 3.19  ±0.18 | 1.10  ±0.05 | 1.11  ±0.25 |
| **TMBB26** | 69  ±2 | 0.46  ±0.02 | 0.30  ±0.00 | 0.85  ±0.04 | 0.55  ±0.00 | 1.56  ±0.09 |
| **TMB3753** | n/a | | | 8.94  ±0.24 | 3.39  ±0.18 | 1.41  ±0.29 |
| **TMBB28** |  | n/a |  | 6.26  ±0.76 | 2.72  ±0.10 | 1.50  ±0.26 |
| **TMBB06** | 57  ±5 | 0.54  ±0.08 | 0.41  ±0.14 | 3.51  ±0.90 | 1.11  ±0.10 | 1.28  ±0.13 |
| **TMBB09** | 71  ±10 | 1.43  ±0.52 | 0.26  ±0.07 | 1.79  ±1.13 | 0.58  ±0.28 | 1.72  ±0.08 |

Fold change values (FC) refer to the difference in fluorescence intensity (FI) after 6h and at the beginning of the experiment (0h) for each cultivation condition. n/a = no subpopulations.

**Table S5.** Normalized fluorescence intensity (FI) data by cell size (FSC) for the *SNF3* deletant *and* wild-type strains on high xylose and low glucose at 6h cultivation

|  | **Normalized FI at 6h** | | | | |
| --- | --- | --- | --- | --- | --- |
| **Strain *SNF3* wt** | **Xylose**  **50 g/L**  **High-FI population** | **Glucose**  **1 g/L** | **Strain**  **s*nf3*Δ** | **Xylose**  **50 g/L**  **High-FI population** | **Glucose**  **1 g/L** |
| **TMB3713** | 0.13 ±0.01 | 0.36 ±0.03 | **TMBB11** | 0.07 ±0.01 | 0.15 ±0.00 |
| **TMB3723** | 0.14 ±0.01 | 0.33 ±0.02 | **TMBB26** | 0.06 ±0.00 | 0.13 ±0.00 |
| **TMB3753** | 0.12 ±0.01 | 0.25 ±0.02 | **TMBB28** | 0.08 ±0.02 | 0.11 ±0.01 |
| **TMBB06** | 0.17 ±0.02 | 0.34 ±0.03 | **TMBB09** | 0.06 ±0.02 | 0.15 ±0.01 |

**Table S6.** Fold change values (FC) for *SNF3*-overexpressing strains TMBB33 (non-xylose metabolizing) and TMBB34 (xylose-metabolizing) across the different cultivation conditions

| **Condition** | **Strain** | **FC** | **Population**  **(%)** |
| --- | --- | --- | --- |
| Xylose 50 g/L | TMBB33* | 13.96 ±0.60 | 34 ±2 |
|  | TMBB34 | 23.84 ±2.78 | Single |
| Glucose 1 g/L | TMBB33 | 15.64 ±0.52 | Single |
|  | TMBB34 | 10.72 ±0.06 | Single |
| No sugar | TMBB33* | 5.57 ±0.02 | 20 ±1 |
|  | TMBB34* | 2.67 ±0.047 | 70 ±1 |
| Glucose 40 g/L | TMBB33 | 2.47 ±0.06 | Single |
|  | TMBB34 | 2.08 ± 0.16 | Single |

FC values correspond to the signal observed at 12 h for TMBB33 and at 10 h for TMBB34 normalized to the corresponding 0h signal. *When two subpopulations appeared under no-sugar or xylose conditions, parameters are reported for the induced subpopulation.


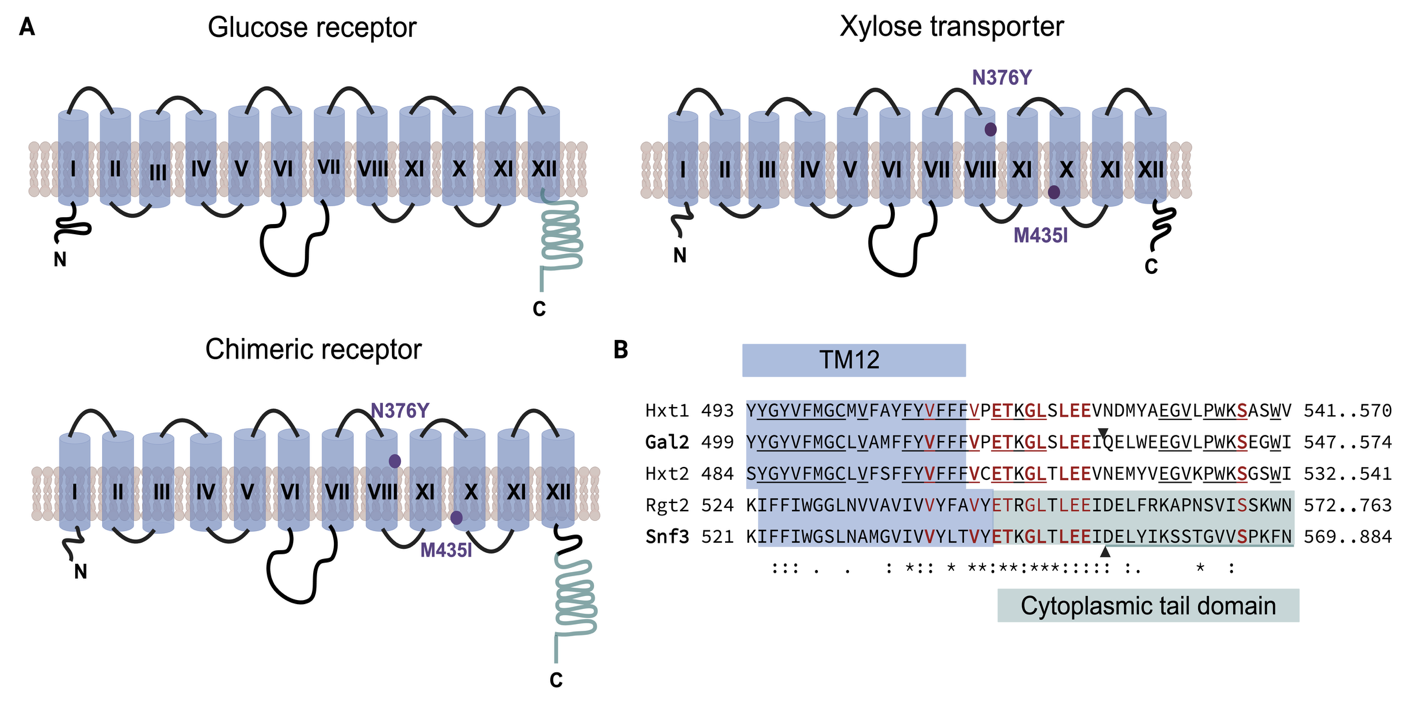
**Figure S3. The chimeric receptor strategy involves assembling specific xylose-binding and signaling domains of a transporter and receptor, respectively.** A) Schematic structures are shown for the mutated Gal2p transporter, the native Snf3p receptor, and the chimeric transporter/receptor. The predicted model of the transporters and sensors comprises 12 transmembrane domains, labeled I to XII (Created with BioRender). Two amino acid substitutions in the galactose permease Gal2p, highlighted in purple, confer xylose specificity characteristics (Rojas et al., 2021a). The hybrid construction combines the transmembrane domains of the Gal2pmut transporter, truncated at the C-terminus (Δ531), with the signaling tail (highlighted in light blue) of Snf3p. B) The protein sequence alignments (Clustal Omega) of hexose transporters (Hxt1p, Hxt2p, and Gal2p) and glucose sensors (Rgt2p and Snf3p) show part of the last transmembrane domain (blue box) and C-terminal regions. The start of the sensing tails of the receptors are marked in the light blue box. Conserved amino acids are shown in red, while residues conserved only among transporters are underlined. The sites of the C-terminal truncation in Gal2p and the Snf3p tail fusion are pointed by the down and up arrows, respectively.

**Table S7.** Parameters of growth and metabolites accumulation of *SNF3* wild-type (TMB3753), *SNF3* deletant (TMBB28) and *SNF3* overexpressing (TMBB34) recombinant *S. cerevisiae* strains during xylose cultivations.

| **Parameter** | **Strain** | |  |
| --- | --- | --- | --- |
|  | **TMB3753** | **TMBB28** | **TMBB34** |
| **Xylose consumption (%)** | 78.1 ±8.3 | 90.3 ±6.0 | 60.6 ±5.7 |
| **Xylitol yield (g_xylitol_/g_xylose_)** | 0.056 ±0.010 | 0.035 ±0.025 | 0.085 ±0.018 |
| **Ethanol yield (g_ethanol_/g_xylose_)** | 0.125 ±0.018 | 0.110 ±0.040 | 0.196 ±0.038 |
| **Acetate yield (g_acetate_/g_xylose_)** | 0.135 ±0.020 | 0.040 ±0.002 | 0.173 ±0.033 |
| **Specific growth rate (µ_max_)** **(h^-1^)** | 0.049 ±0.001 | 0.054 ±0.001 | 0.043 ±0.003 |
